# Circular MIMO antenna with ML-based bandwidth and isolation prediction for 6G communications

**DOI:** 10.1038/s41598-026-54274-w

**Published:** 2026-05-25

**Authors:** Md Mahabub Alam, Messaoud Ahmed Ouameur, Md Ershadul Haque, Md. Ashraful Haque, Jun-Jiat Tiang, Narinderjit Singh Sawaran Singh, Ruwaybih Alsulami, Saeed Alzahrani

**Affiliations:** 1 Faculty of Electrical and Electronics Engineering Technology, Universiti Malaysia Pahang Al-Sultan Abdullah, 26600 Pekan, Pahang Malaysia; 2 Department of Electrical Engineering and Computer Engineering, Université du Québec à Trois-Rivières, Léon-Provancher, Trois-Rivières, QC 2465 Canada; 3 School of Computing Mathematics and Engineering, Charles Sturt University, Bathurst, NSW 2795 Australia; 4 Department of Electrical and Electronic Engineering, University of Liberal Arts Bangladesh (ULAB), Dhaka, 1207 Bangladesh; 5 Centre for Wireless Technology, CoE for Intelligent Network, Faculty of Artificial Intelligence and Engineering, Multimedia University, Persiaran Multimedia, 63100 Cyberjaya, Selangor Malaysia; 6 Faculty of Data Science and Information Technology, INTI International University, Nilai, Malaysia; 7 Department of Electrical Engineering, Umm Al-Qura University, 24211 Makkah, Saudi Arabia; 8 Department of Electrical Engineering, University of Tabuk, 71491 Tabuk, Saudi Arabia

**Keywords:** Machine learning, MIMO antenna, Bandwidth, THz antenna, Isolation, 6G, Industrial and Innovation, Engineering, Mathematics and computing

## Abstract

The demanding requirements of next-generation 6G wireless systems necessitate the development of compact, wideband, and high-isolation terahertz (THz) MIMO antennas, while conventional full-wave electromagnetic optimization remains computationally expensive for complex multi-parameter designs. To overcome these challenges, this work introduces a compact circular MIMO antenna integrated with a machine learning (ML)-based framework for efficient performance prediction and design optimization. The proposed antenna consists of two co-oriented circular radiating elements, enhanced with a concentric ring and side stubs to improve impedance matching and broaden bandwidth. High inter-element isolation is achieved through the incorporation of isolation walls and an optimized partial ground structure. The polyimide-based antenna, with compact dimensions of 130 × 70 μm², operates at 5.55 THz and provides a wide bandwidth of 4.56–5.86 THz, a peak gain of 8.04 dB, a radiation efficiency of 85.64%, a diversity gain (DG) of 9.985 dB, and a total active reflection coefficient (TARC) below − 35 dB. To enable rapid performance estimation, an ML-based predictive model employing five supervised regression algorithms is trained using crucial geometrical parameters, including inner ring radius, feedline width, stub width, element spacing, and substrate height. Among the evaluated models, Cat Boost regression achieves the highest prediction accuracy, with R² scores of 97.05% for bandwidth and 92.56% for isolation. These results demonstrate that the proposed circular MIMO antenna, supported by ML-based predictive modeling, offers a promising solution for compact, high-performance antennas in 6G THz communication systems.

## Introduction

Terahertz (THz)-band communications are widely regarded as a key enabler for meeting the extraordinary data rate demands of sixth-generation (6G) wireless networks and next-generation mobile systems. This potential stems from the availability of vast, largely underutilized spectrum resources at THz frequencies^1,2^. In recent years, significant research efforts have focused on millimeter-wave (mm-Wave) systems operating in the 30–300 GHz frequency range. However, due to the limited available bandwidth, typically only a few tens of GHz, achieving terabit-per-second data rates at mm-Wave frequencies requires extremely high physical-layer spectral efficiencies, often reaching several hundred bits per second per Hertz^3,4^. In contrast, the THz spectrum (0.3–10 THz) offers bandwidths extending into the hundreds of GHz, enabling terabit-per-second data rates with substantially lower spectral efficiency requirements^5,6^. Moreover, emerging 6G use cases, including immersive Extended Reality (XR), holographic communications, and large-scale Internet of Things (IoT) deployments, demand unprecedented bandwidth resources. Beyond communications, the THz band also supports a wide range of applications such as high-resolution imaging for security screening, non-destructive material characterization, biomedical sensing and diagnostics, and ultra-fast wireless backhaul links. These diverse applications position the THz band as a strong candidate to complement or even replace mm-wave technologies in future wireless and sensing systems^7,8^. Nevertheless, the advantages of ultra-wide bandwidth at THz frequencies are accompanied by severe propagation losses, molecular absorption, and atmospheric attenuation, which significantly limit communication range and reliability^9,10^. As a result, advanced antenna system designs are indispensable to fully exploit the bandwidth potential of THz communications while mitigating propagation impairments.

To address these challenges, conventional single-element microstrip antennas have been widely studied for THz communication systems due to their simple structure, low profile, and ease of fabrication. However, they suffer from limited gain, narrow bandwidth, and low data throughput, making them insufficient for next-generation 6G requirements^11^. To enhance performance, various techniques have been explored, among which slotting methods are particularly effective in improving impedance bandwidth, gain, and radiation characteristics without increasing antenna size. Other approaches, such as parasitic elements, metamaterials, defected ground structures (DGS), and dielectric loading, can also enhance performance but often introduce additional complexity and fabrication challenges^12^. To overcome the inherent limitations of single-element antennas, recent research has increasingly focused on multiple-input multiple-output (MIMO) antenna systems for THz communications. By exploiting spatial diversity and multiplexing gains, MIMO technology significantly enhances signal robustness, spectral efficiency, and channel capacity compared to single-antenna configurations, making it a key enabler for high-speed wireless systems^13^. Microstrip-based MIMO antennas, particularly those incorporating slotted configurations, offer a balanced solution by combining structural simplicity with enhanced electromagnetic performance. Their compact form factor, low profile, and compatibility with planar fabrication processes make them highly suitable for large-scale integration at THz frequencies^14^. Furthermore, the extremely short wavelengths at THz bands enable dense antenna packing, facilitating high-capacity MIMO systems within compact and lightweight platforms, such as mobile, wearable, and on-chip devices^15,16^. Despite these advantages, conventional rectangular MIMO array configurations often exhibit increased spatial correlation and directional radiation patterns, which can degrade performance in multipath-rich environments^17^. In contrast, circular MIMO array geometries provide uniform 360° angular coverage, reduced mutual coupling, and improved spatial diversity, resulting in enhanced system performance in complex propagation scenarios^18^. Therefore, circular slotted microstrip MIMO antenna designs represent a promising and efficient solution for meeting the demanding requirements of 6G THz communication systems.

Recent MIMO antenna research has pursued diverse structural strategies to simultaneously enhance bandwidth, gain, and diversity metrics. In^19^, a THz antenna operating over the 4.03–10 THz band is designed on a polyimide substrate (90 × 30 μm²), supporting both single-element and two-port MIMO configurations for 6G applications, achieving 12.38 dB gain, 89% efficiency, and − 27 dB isolation. The design further demonstrates excellent diversity performance with ECC = 0.002 and DG = 9.99, while an equivalent RLC circuit model accurately characterizes the S₁₁ response. Reference^20^ presents a proximity-coupled graphene patch-based two-port THz MIMO antenna with pattern diversity, operating over 1.76–1.87 THz with a 10 dB impedance bandwidth of 6.06%. The design achieves isolation > 25 dB and maintains acceptable ECC, DG, MEG, and TARC values, while graphene-based tunability enables controlled single-mode operation and flexible THz performance. The design in^21^ presents a tri-band graphene-based series-fed microstrip two-element THz MIMO antenna operating at 2.3, 3.2, and 4.5 THz, achieving bandwidths of 38, 43, and 60 GHz, respectively. It provides isolation > 15 dB, gain above 5 dBi, ECC < 0.2, and near-ideal diversity gain, demonstrating reliable MIMO performance for THz communication systems. In^22^, a wideband THz pixel-based multiple-slot patch antenna on FR4 achieves a 0.59–0.91 THz bandwidth (0.32 THz, 42.67% fractional bandwidth) through optimized cavity structures on the radiating surface. The design offers 4.04 dBi gain and 70% efficiency, demonstrating suitability for wideband THz communication and material characterization applications.

Although researchers broadly target bandwidth, gain, and efficiency improvements, mutual coupling remains a foundational challenge in MIMO antenna arrays. Reducing inter-element spacing intensifies electromagnetic cross-talk, elevating signal correlation and degrading system performance. Maintaining at least λ/2 separation or preferably λ/4 for stronger decoupling, keeps isolation below − 15 dB, yielding lower ECC, improved diversity gain, and reduced CCL, motivating extensive investigation into structural and material-level coupling suppression. The design in^23^ addresses this through a slotted ground plane, achieving 2.5 THz bandwidth (6.2–8.7 GHz), 14.59 dB peak gain, a 100 × 300 μm² footprint, isolation exceeding − 31 dB, and 96% radiation efficiency. Among six supervised regression models evaluated using variance score, R², MSE, MAE, and RMSE, Extra Trees Regression delivers the lowest prediction error. In^24^, a polyimide substrate with a graphene interelement barrier targets 6G THz bands, spanning 4.331 THz (0.631–4.962 THz) within a 95.52 × 227.24 μm² footprint. The design achieves ~ 27 dB isolation, 13.3 dB gain, 95% efficiency, ECC below 0.0002, and diversity gain exceeding 9.99. An RLC equivalent circuit developed in ADS is validated against CST MWS simulations, and Gaussian Process Regression attains ~ 99% predictive accuracy. Reference^25^ employs a separation wall technique on a 160 × 160 μm² polyimide substrate, achieving dual-band operation across 0.081–1.36 THz and 1.81–3.43 THz with gains of 11.91 and 12.21 dB and isolation of 31.43 and 36.1 dB respectively. CST-based design is corroborated through ADS RLC modeling, while XGBoost Regression delivers over 96% accuracy in gain prediction, supporting its applicability to compact 6G MIMO systems.

Beyond mutual coupling mitigation, the escalating complexity of THz MIMO antenna design has increasingly driven machine learning (ML) and artificial intelligence (AI) integration across performance prediction, automated synthesis, and intelligent design optimization^24–30^. Unlike conventional electromagnetic (EM) simulation-based approaches, which, despite their accuracy, are computationally intensive and time-consuming for high-dimensional design spaces, ML methods offer speed improvements of up to three to seven times while effectively handling nonlinear and multimodal design characteristics. By leveraging data-driven surrogate models, ML replaces repeated EM simulations with fast and reliable approximations, enabling efficient exploration of large design spaces at reduced computational cost. These models also facilitate accurate prediction of key performance metrics, including gain, isolation, bandwidth, and efficiency. Moreover, techniques such as deep neural networks and reinforcement learning leverage large datasets to enable faster, more efficient optimization, effectively handling high-dimensional design spaces and multi-objective performance requirements compared to conventional methods. In^26^, the authors propose a compact polyimide-based THz MIMO antenna (0.614λ₀ × 0.614λ₀) achieving 15 dB gain, > 31 dB isolation, 97.4% efficiency, ECC < 0.0012, and 85.71% bandwidth. A Random Forest Regression model provides > 95% prediction accuracy, closely matching CST results. Similarly^27^, applies supervised ML models for THz MIMO antennas, where Gradient Boosting Regression achieves over 94% accuracy in isolation prediction with strong agreement with simulations. In^28^, a compact four-element THz MIMO antenna demonstrates multi-band operation with high gain (13.35 dB), strong isolation (− 34.04 dB), and excellent efficiency (96.04%), making it suitable for 6G high-data-rate applications. Random Forest Regression achieves high prediction accuracy (R² = 93.93%) for antenna gain, closely matching CST simulation results and enabling efficient performance estimation. In^29^, a regression-based ML framework is proposed for a dual circular ring graphene FSS absorber, achieving 99.9% absorption at 10.5 THz with a 1 THz bandwidth (10.0–11.0 THz). Among nine evaluated models, the Random Forest Regressor shows the best performance with R² ≈ 1 and high prediction accuracy. In^30^, an ML-based framework is proposed for THz antenna design to overcome the high computational cost of conventional methods. Models including KNN, XGBoost, Decision Tree, and Random Forest are used to predict return loss. Among them, Random Forest achieves the best performance with 82% accuracy and an MSE of 3.816. The results demonstrate the potential of ML for efficient and optimized 6G THz antenna design. Despite these advances, ML applications in circular THz MIMO antenna design remain limited, highlighting opportunities for future research in ML-driven optimization and advanced decoupling techniques for 6G systems.

This study addresses several crucial innovations and contributions which are as follows:

i. Novel circular MIMO antenna design on polyimide substrate: A novel circular MIMO antenna on polyimide substrate surpasses conventional rectangular designs through its symmetric radiation profile, uniform inter-element spacing, and suppressed surface current concentration, inherently reducing ECC and enhancing diversity gain. The polyimide substrate’s low dielectric loss and thermal stability further support wider impedance bandwidth and superior THz efficiency within a compact footprint. The antenna demonstrates exceptional performance, resonating at 5.55 THz with a return loss of − 48.32 dB, 1.30 THz bandwidth, 8.038 dB gain, ultra-low ECC of 0.0035, and diversity gain of 9.985 dB.
ii. Fast Optimization Using ML Techniques: Supervised regression-based ML techniques have emerged as a compelling alternative to computationally intensive full-wave simulations, training on simulation-derived datasets to capture complex relationships between geometric variables and antenna performance. Models including Random Forest, Extra Trees, XGBoost, Decision Tree, and CatBoost are employed to accurately predict bandwidth and isolation across the 3–8 THz range, facilitating rapid design iteration and intelligent optimization for next-generation 6G MIMO systems.
iii. Isolation improvement using partial grounding and wall techniques: The two-element MIMO configuration employs a 7 μm copper isolation wall alongside an optimized partial ground structure, maintaining 60 μm inter-element separation to redirect surface currents and suppress mutual coupling to − 23 dB.

## Design methodology

The proposed antenna is designed using a polyimide substrate due to its suitability for terahertz (THz) applications. Polyimide offers low dielectric loss and stable electrical properties at high frequencies, which help minimize signal attenuation and support wideband performance. Its excellent thermal stability ensures reliable operation under high-frequency conditions, while its mechanical flexibility and thin profile enable compact and lightweight antenna configurations. In addition, compared to conventional high-frequency substrates such as Rogers RT/Duroid 5880, polyimide provides a cost-effective and scalable alternative without compromising EM performance. Building on these material advantages, the following section traces the evolutionary development of the proposed circular MIMO antenna, from foundational single-element functionality through to the advanced high-performance MIMO configuration.

### Evolution of the proposed circular MIMO antenna

**Fig. 1:**
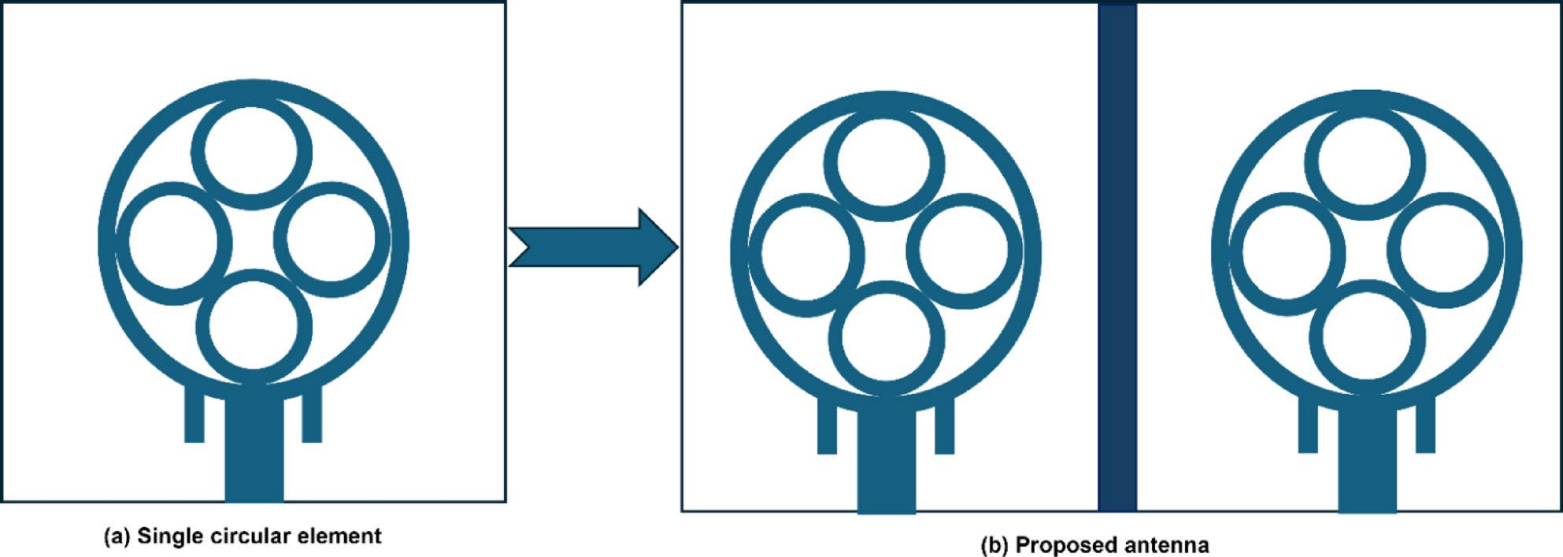
Structural layout of (**a**) Single circular element (**b**) Proposed circular MIMO antenna.

As illustrated in Fig. 1a,b, the proposed antenna evolves systematically from a single circular radiating element to a compact two-port MIMO configuration. The initial element consists of a circular ring loaded with four inner loops, chosen to exploit the intrinsic advantages of circular geometry, such as symmetric radiation characteristics and dual-polarization capability. This dual-polarization behavior originates from the structural symmetry, which supports two orthogonal degenerate modes at the same resonant frequency. When excited through orthogonal feed orientations, independent electric field components (E_x_ and E_y_) are generated, enabling polarization diversity and enhanced channel capacity.

Building upon this single-element foundation, the MIMO configuration is realized on a polyimide substrate ($\:{{\upepsilon\:}}_{r}$ = 3.5, tan δ = 0.0027) backed by a high-conductivity copper ground plane to ensure low-loss terahertz operation. As shown in Fig. 1, two identical circular patch elements are then arranged side by side with identical orientation and a precisely defined center-to-center spacing of 60 μm. This configuration is carefully selected to minimize spatial correlation while maintaining strong isolation between the ports. The resulting two-port circular MIMO antenna achieves a compact footprint of 130 μm × 70 μm, effectively accommodating the dual-element arrangement without compromising isolation or radiation performance. The symmetric placement and orientation of the elements contribute to uniform radiation behavior and reduced mutual coupling, forming a robust foundation for high-performance THz MIMO operation.

## Design of proposed single-element circular antenna

**Fig. 2:**
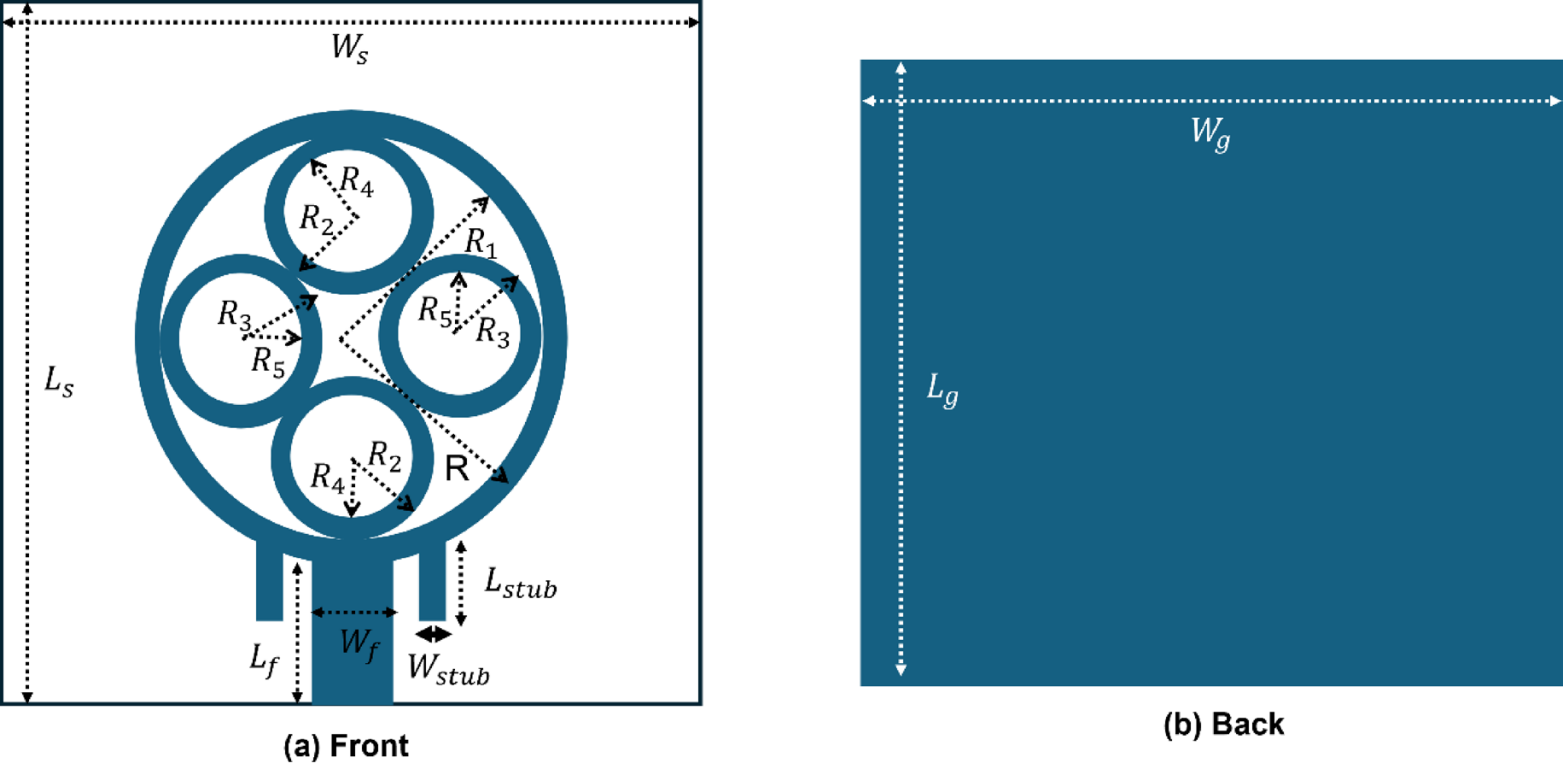
Single circular antenna element: (**a**) Front and (**b**) Back configuration.

The structure shown in Fig. 2 is selected based on established circular microstrip resonator and partial ground-plane antenna design principles to achieve compact size and wideband operation in the THz frequency range. The circular geometry is first theoretically designed using the standard cavity model of a circular microstrip patch antenna. In this approach, the initial patch radius (a) is calculated using Eq. (1), which is derived from the dominant TM₁₁ mode resonance condition^31^.

1 $$\:a=\frac{F}{{\{1+\frac{2h}{\pi\:{\epsilon\:}_{r}F\:}\:[\mathrm{ln}\left(\frac{\pi\:F}{2h}\right)+1.7726\left]\right\}}^{\frac{1}{2}}}$$

Here, h is the substrate thickness, $\:{\epsilon\:}_{r}$ is the relative permittivity of the substrate material, and F is a derived constant that makes the formula for determining the patch radius a simpler. The resonant behavior is governed by Eq. (2)^32^.

2 $$f_{r} = \frac{{x_{{11}} *c}}{{2\pi f_{r} }} = \frac{{1.8412*c}}{{2\pi f_{r} }}$$

where $\:{f}_{r}$ is the resonant frequency or working frequency, the first zero of $\:{{\boldsymbol{J}}_{1}}^{{\prime\:}}$ for TM₁₁ is $\:{x}_{11}$≈1.8412 and c represents the speed of light (3 × 10^8 m/s).

Using Eqs. (1) and (2), the initial radius a is determined from the chosen operating frequency, substrate height, and dielectric constant, providing a physically based starting point for the antenna design. The geometry is then refined using full-wave electromagnetic simulations in CST Microwave Studio to account for practical effects such as fringing fields, coupling, and fabrication constraints. The circular ring structure is selected due to its symmetrical current distribution and ability to support multiple resonant modes, which enhances bandwidth. A partial ground plane is employed to improve impedance matching by modifying surface current distribution and strengthening coupling between the feedline and radiating element. Additionally, embedded circular slots are introduced to perturb current paths and generate extra resonant modes, further improving bandwidth and radiation performance. All parameters are finally optimized through systematic parametric analysis to achieve an optimal balance between compactness, impedance matching, and overall antenna performance.

Feeding a THz antenna involves significant challenges due to the extremely high operating frequency and submicron dimensional constraints. At 5.55 THz, conventional coaxial or SMA connectors are not suitable due to excessive physical size and high insertion losses, necessitating fully planar or on-chip feeding techniques. In the proposed design, a microstrip feedline is employed with optimized dimensions to achieve 50 Ω impedance matching, ensuring efficient power transfer to the radiating element while remaining compatible with planar nanofabrication processes. At the device integration level, coplanar waveguide (CPW) feeds and integrated waveguide transitions are commonly used alternatives, enabling direct coupling between the antenna and THz integrated circuits. From a system perspective, THz excitation can be realized using photonic and electronic sources such as photo-mixers based on optical heterodyning, quantum cascade lasers (QCLs), or photoconductive antennas. These sources can deliver THz signals either through direct on-chip integration or via near-field and waveguide coupling mechanisms, depending on the system architecture. The use of a low-loss polyimide substrate further enhances feeding efficiency by minimizing dielectric attenuation and supporting stable signal propagation at THz frequencies. Collectively, these feeding strategies ensure practical excitation of the proposed antenna for high-frequency THz MIMO applications (Table 1).

**Table 1:** The optimized single element circular antenna dimensions.

Design Parameter	Symbol	Optimized dimension [µm]	Design Parameter	Symbol	Optimized dimension [µm]
Substrate width /Ground width	$\:{W}_{s}/{W}_{g}$	70.00	Inner radius of large circle	$\:{R}_{1}$	18
Substrate length	$\:{L}_{s}$	70.00	Outer and Inner radius of small circle 1 and 3	$\:{R}_{2}$, $\:{R}_{4}$	8,6
Ground length	$\:{L}_{g}$	60.00	Outer and Inner radius of small circle 2 and 4	$\:{R}_{3}$, $\:{R}_{5}$	7,5
Width of feedline	$\:{W}_{f}$	6.00	Rectangular stub of length	$\:{L}_{stub}$	7.28
Length of feedline	$\:{L}_{f}$	13.23	Rectangular stub of width	$\:{W}_{stub}$	2
Metal thickness	*t*	0.5	Substrate thickness	h	11
Outer radius of large circle	*R*	20			

The proposed single-element circular antenna is designed on a compact rectangular substrate with lateral dimensions Wₛ × Lₛ = 70 μm × 70 μm a dielectric thickness of h = 11 μm, and a metallization thickness of t = 0.5 μm, as summarized in Table 1. A ground plane of dimensions $\:{W}_{g}$ × $\:{L}_{g}$= 70 μm × 60 μm is placed beneath the substrate. The reduced ground plane length ($\:{L}_{g}$< *L*_*s*_) forms a partial ground configuration, which improves impedance matching and enhances bandwidth by modifying the surface current distribution and strengthening the coupling between the feedline and the radiating element. The radiating structure consists of a circular ring element with an outer radius of *R* = 20 μm and an inner radius of R_1_ = 18 μm, resulting in a ring width of 2 μm. To further enhance performance, four symmetrically embedded circular slots are introduced within the ring. These slots are arranged in two pairs: one with radius R₂, $\:{R}_{4}$= 8, 6 μm and the other with radius R₃, R_5_ = 7, 5 μm. This configuration creates multiple resonant paths, improving impedance matching and broadening the operational bandwidth while enhancing radiation efficiency. The antenna is excited using a microstrip feedline of dimensions $\:{W}_{f}$×$\:{L}_{f}$=6.00 μm×13.23 μm. A rectangular impedance-matching stub ($\:{L}_{stub}$×$\:{W}_{stub}$=7.28 μm×2 μm) is incorporated at the feed junction to ensure efficient power transfer and minimize reflection losses.

## Analysis of single circular element

### Design approach and performance evaluation

**Fig. 3:**
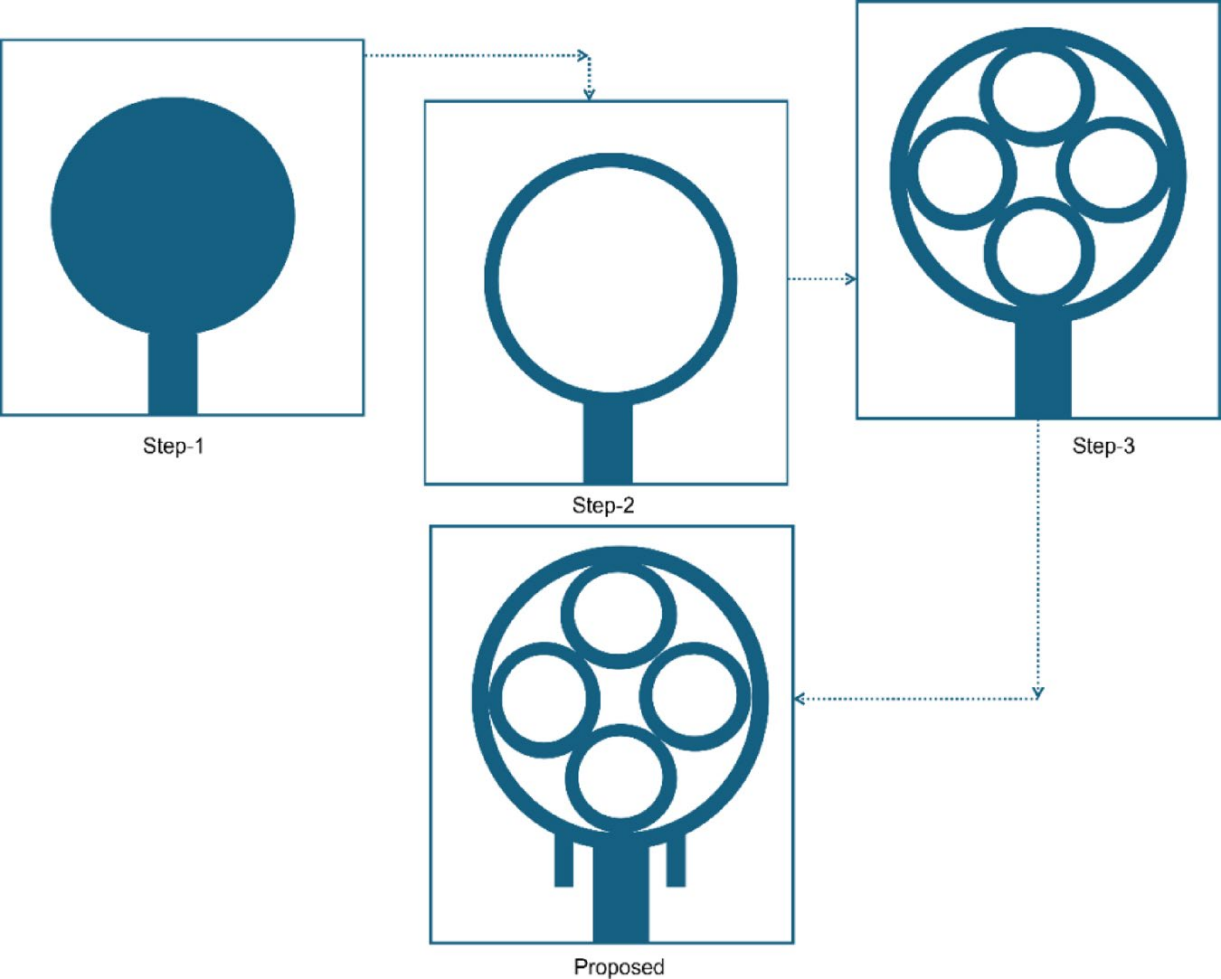
Methodological steps in single-element circular antenna design.

The design process of the single-element circular antenna involved a sequence of four key stages to achieve the intended performance. The initial structure, shown in Fig. 3, did not provide satisfactory results and was considered a failure due to the excessive enhancement and near elimination of return loss. The second iteration introduced a circular ring architecture to address these deficiencies, while the third stage incorporated four smaller circular slots embedded within a larger circular patch, yielding improved performance characteristics that approached but did not fully satisfy the design objectives. The final optimization phase integrated two stubs with the preceding configuration, culminating in an optimized antenna structure that successfully achieved favorable performance metrics. This progressive design methodology, characterized by iterative modifications and performance refinements, ultimately validated the effectiveness of the systematic approach in developing a high-performance circular patch antenna suitable for 6G THz application requirements.

**Fig. 4:**
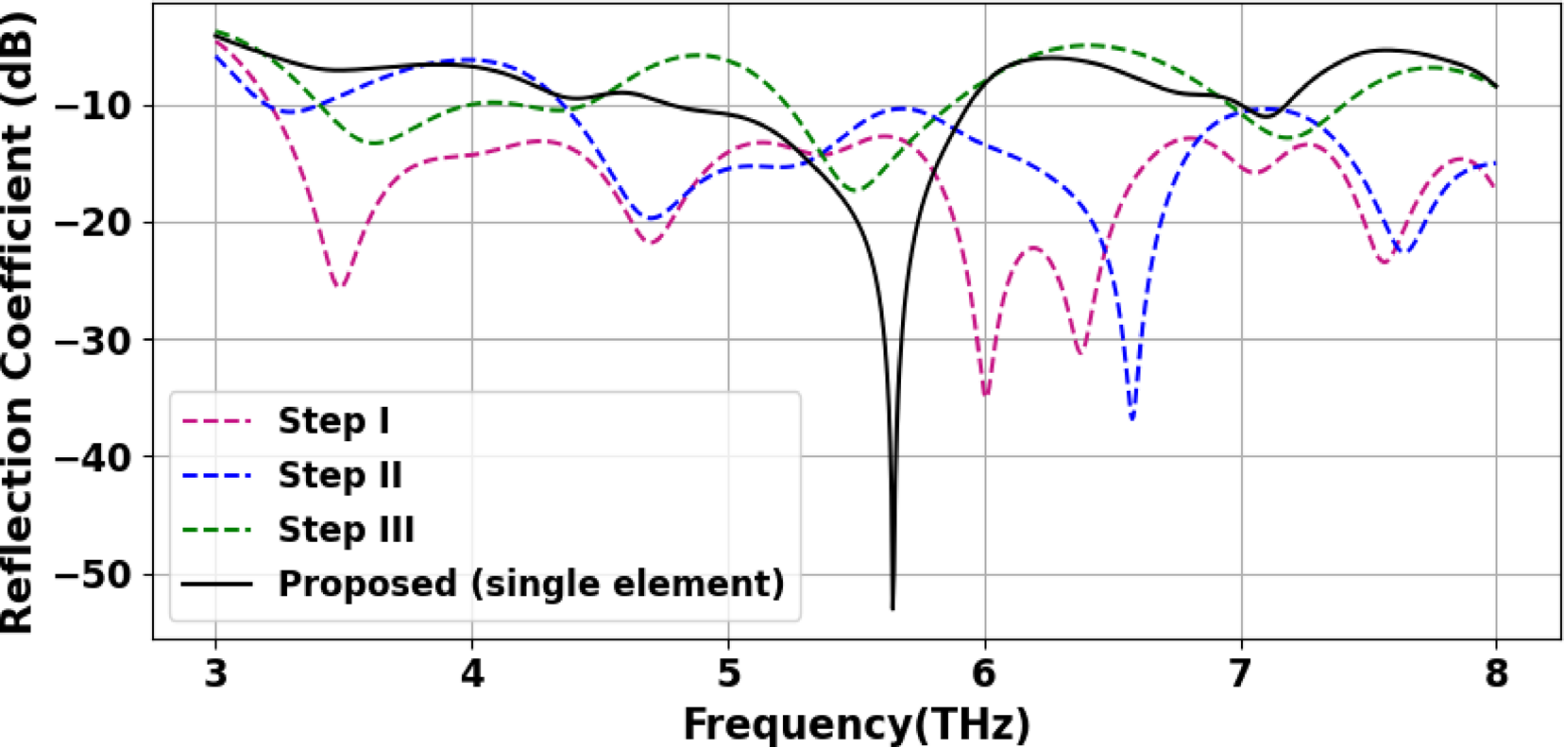
Stepwise reflection coefficient of single-element circular antenna.

Figure 4 presents the simulated four-stage evolution of the circular antenna design, where each successive modification results in observable changes in the reflection coefficient (S₁₁), resonant frequency, and impedance bandwidth. The first stage establishes the foundational configuration including a basic circular patch fed by a microstrip line. This baseline design exhibits multi-band operational behavior across the 3–8 THz range. However, none of the resonant bands achieve satisfactory return loss values, with S₁₁ remaining well above the acceptable − 10 dB threshold. The bandwidth at each resonance is moderate but insufficient for reliable operation, indicating that while the circular geometry supports multi-band excitation, the feeding mechanism and patch dimensions require optimization. The second design iteration introduces a structural modification, specifically tailored to enforce dual-band resonance, yielding two distinct resonant frequencies at 4.7 THz and 6.58 THz. Although this represents a significant improvement in frequency selectivity compared to the first stage, the return loss values at both bands remain suboptimal, failing to achieve the desired − 10 dB criterion. Moreover, the higher-frequency band at 6.58 THz exhibits a notably narrow bandwidth, which would limit practical applicability in wideband terahertz systems. This stage confirms that dual-band operation can be achieved through geometric modification, but further refinement is needed to improve impedance matching. In the third design phase, an additional structural modification is introduced to enhance performance, but it instead leads to degradation. The antenna exhibits three resonances at 3.62 THz, 5.5 THz, and 7.2 THz, suggesting disruption of the original resonant behavior and the emergence of new modes. Despite the broader spectral coverage, the S₁₁ values at all resonances remain above − 10 dB, making this configuration impractical. This result highlights the sensitivity of the circular patch geometry to structural changes and emphasizes the need for systematic optimization rather than arbitrary adjustments. The fourth and final stage applies targeted dimensional refinements based on insights from earlier iterations, resulting in a clear performance improvement. The antenna achieves a single, well-defined resonance at 5.64 THz with an excellent return loss of − 52.91 dB, far exceeding the − 10 dB criterion. The bandwidth is also satisfactory, indicating efficient impedance matching. This confirms that focusing on a single optimized band yields better performance than the multi-resonance designs, making this configuration the optimal choice for advanced THz applications.

### Parametric study of single-element circular antenna results

#### Performance analysis with varying feed width ($\:{W}_{f}$)

**Fig. 5:**
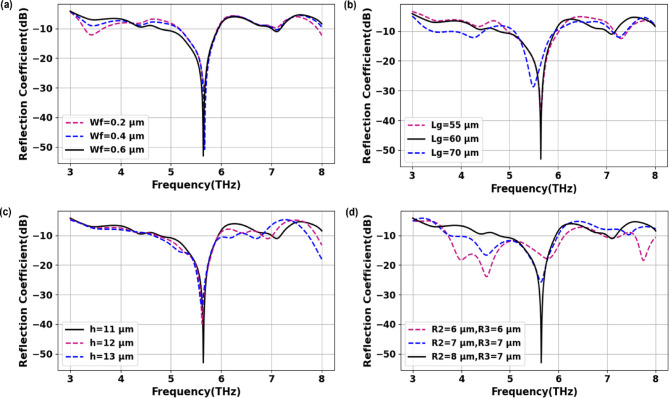
Parametric analysis results showing variation of (**a**) Width of feedline ($\:{W}_{f}$), (**b**) Length of ground ($\:{L}_{g}$), (**c**) Substrate height (**h**), and (**d**) Outer radii of small circles ($\:{R}_{2}$, $\:{R}_{3}$).

Figure 5a presents a parametric analysis of the feed line width (W_f_) at 0.2 μm, 0.4 μm, and 0.6 μm to evaluate its effect on impedance matching. The selected step sizes are chosen to represent practical sub-micron fabrication variations and to sufficiently capture the impedance transition behavior within the available design space. As observed, W_f_ variations do not affect the resonant frequency, which remains stable at 5.55 THz, since resonance is primarily governed by the radiating patch dimensions and substrate properties. However, W_f_ significantly influences impedance matching, reflected in the depth of the S11 response. At W_f_ = 0.2 μm, poor matching is observed with a shallow return loss. Improvement is seen at 0.4 μm, while the optimum performance occurs at 0.6 μm with a minimum return loss of − 52.91 dB, attributed to better impedance matching between the feed line and patch due to reduced characteristic impedance. A local sensitivity observation indicates that small variations around the optimum value (± 0.1 μm) have negligible impact on resonant frequency and only minor effect on return loss, confirming stable performance around the selected design point. Therefore, Wf = 0.6 μm is selected as the optimal value, balancing impedance matching performance and fabrication feasibility at THz-scale dimensions.

#### Ground plane length effects on antenna performance

The parametric investigation of ground plane length (55 μm, 60 μm, and 70 μm) is carried out to quantify its influence on impedance matching and to justify the selection of the optimal design value. The chosen step variations are selected to represent realistic fabrication tolerances at the sub-micron scale while ensuring sufficient resolution to capture performance trends. As shown in Fig. 5b, all configurations maintain stable single-band operation around 5.6 THz, indicating that the resonant frequency is primarily governed by the radiating patch rather than the ground plane. However, the ground plane length significantly affects impedance matching and return loss behavior. At 55 μm, suboptimal matching is observed, while 70 μm yields moderate performance with a return loss of approximately − 30 dB. The 60 μm configuration provides the best impedance matching, achieving a resonant frequency of 5.64 THz and an improved return loss of − 52.92 dB. A local sensitivity observation indicates that small deviations around the optimal value (± 5 μm) result in limited variation in resonant frequency but noticeable degradation in return loss, confirming that 60 μm represents a stable and robust operating point. Therefore, 60 μm is selected as the optimal ground plane length, balancing impedance matching performance and fabrication feasibility for terahertz applications.

#### Parametric study of substrate height (h)

Figure 5c presents a parametric study of substrate height (*h*) across three values,11 μm, 12 μm, and 13 μm, to examine its influence on the antenna’s impedance matching and return loss characteristics. As seen in the figure, varying *h* does not produce a shift in the resonant frequency, which remains fixed at 5.5 THz across all three configurations. This behavior is expected, as the resonant frequency of a circular patch is primarily governed by the patch radius and the substrate’s dielectric constant rather than its thickness. However, the substrate height has a clear effect on the depth and sharpness of the S₁₁ resonance notch. The 11 μm configuration yields the deepest return loss, indicating superior impedance matching and a higher resonance quality factor compared to the 12 μm and 13 μm cases. As h increases beyond 11 μm, the return loss degrades, suggesting increased fringing fields and impedance mismatch. Therefore, 11 μm is selected not for tuning the resonant frequency, but for achieving the best trade-off between structural feasibility and electromagnetic performance within polyimide-based terahertz antenna constraints. This confirms that while h does not control the resonant frequency, it is critical for optimizing return loss and ensuring stable resonance behavior.

#### Effect of embedded circle radii (R₂, R₃) on circular patch antenna

Figure 5d illustrates the parametric analysis of embedded circle radii (R₂, R₃) through systematic evaluation of three distinct configurations: (R₂=6 μm, R₃=6 μm), (R₂=7 μm, R₃=7 μm), and (R₂=8 μm, R₃=7 μm), demonstrating their profound impact on antenna electromagnetic characteristics. The investigation reveals that the asymmetric configuration (R₂=8 μm, R₃=7 μm) delivers superior performance metrics, exhibiting enhanced reflection coefficient behavior, expanded operational bandwidth, and improved radiation efficiency compared to symmetric radius configurations. The parametric study demonstrates that variations in embedded circle radii significantly influence resonance depth characteristics and frequency stability parameters, establishing the critical importance of precise circular patch tuning for achieving optimal antenna impedance matching and bandwidth control.

## Circular MIMO structure

Data transmission speeds in THz communication, operating within the 0.1–10 THz frequency band^33^, are several orders of magnitude higher than those of traditional sub-6 GHz wireless systems. Circular MIMO antennas are preferred over single-element circular antennas because they enable the simultaneous transmission of multiple data streams, significantly enhancing spectral efficiency, system throughput, and overall data rates. Furthermore, by delivering superior spatial diversity, polarization diversity, and radiation symmetry compared to conventional rectangular MIMO architectures, the integration of circular MIMO antenna technology into the THz domain marks a transformative step in the evolution of wireless communication^34^.

### Proposed circular MIMO antenna

**Fig. 6:**
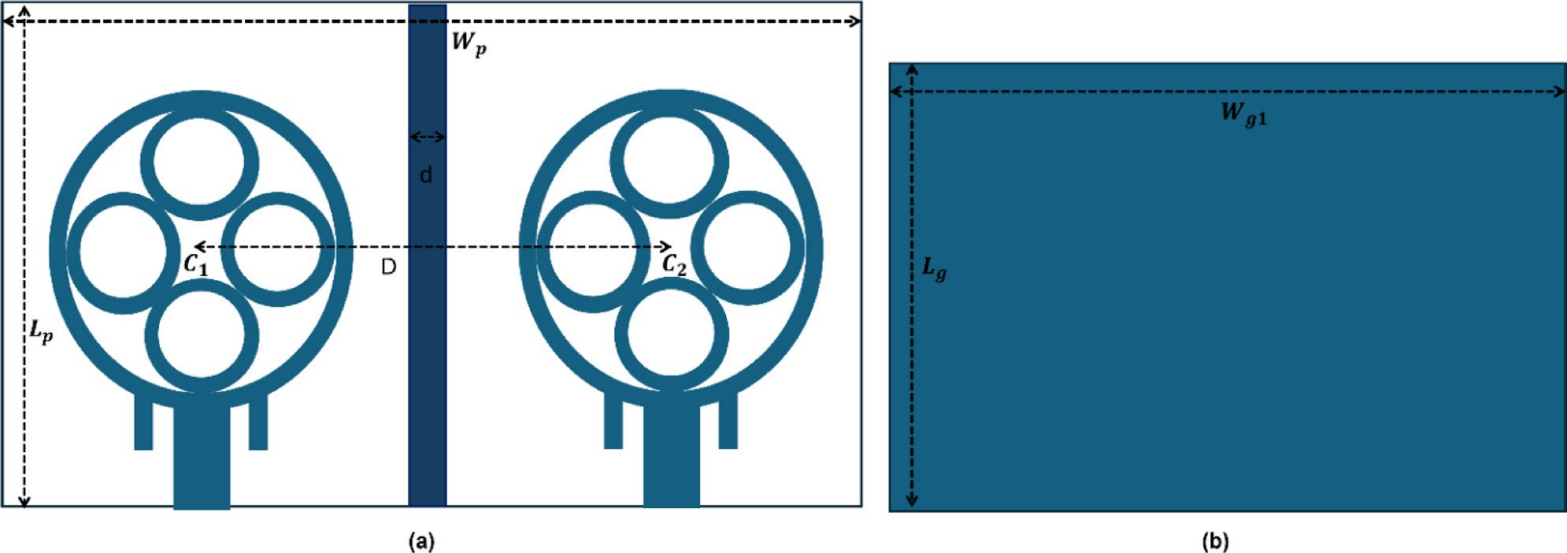
(**a**) Front and (**b**) Back view of proposed circular MIMO antenna.

Figures 6a,b illustrate the front and back views of the proposed circular MIMO antenna in a 1 × 2 configuration, comprising two transmit and two receive elements. The circular patch radiators are positioned at an optimized center-to-center spacing of D = 60 μm, determined through rigorous parametric analysis to minimize mutual coupling and enhance antenna performance. To further improve isolation, a copper barrier with width d = 7 μm and height h = 11 μm is introduced between the radiating elements. The chosen width allows the barrier to fit precisely within the inter-element gap between the two circular patch radiators without overlapping their boundaries, while the height corresponds to the full substrate thickness, enabling it to span the entire dielectric layer and maximize shielding effectiveness against transverse electromagnetic leakage. Parametric sweeps conducted over a range of d = 3–15 μm confirm that d = 7 μm provides the optimal trade-off between physical feasibility and isolation enhancement, achieving maximum suppression of electromagnetic coupling at the target 3–8 THz operating frequency while preserving the radiation characteristics of each element. The compact ground plane, shown in Fig. 6b, measures W_g1_ = 130 μm in width and L_g_ = 60 μm in length and incorporates a rectangular defected ground structure (DGS) that, consistent with the single-element design, disrupts surface current distribution to enhance port isolation and mitigate coupling. This integrated design approach achieves robust MIMO performance while maintaining an ultra-compact footprint, ensuring minimized envelope correlation coefficient (ECC), enhanced isolation, and optimized radiation characteristics, critical for 6G THz communication.

### Performance of barrier walls in circular MIMO antenna isolation

**Fig. 7:**
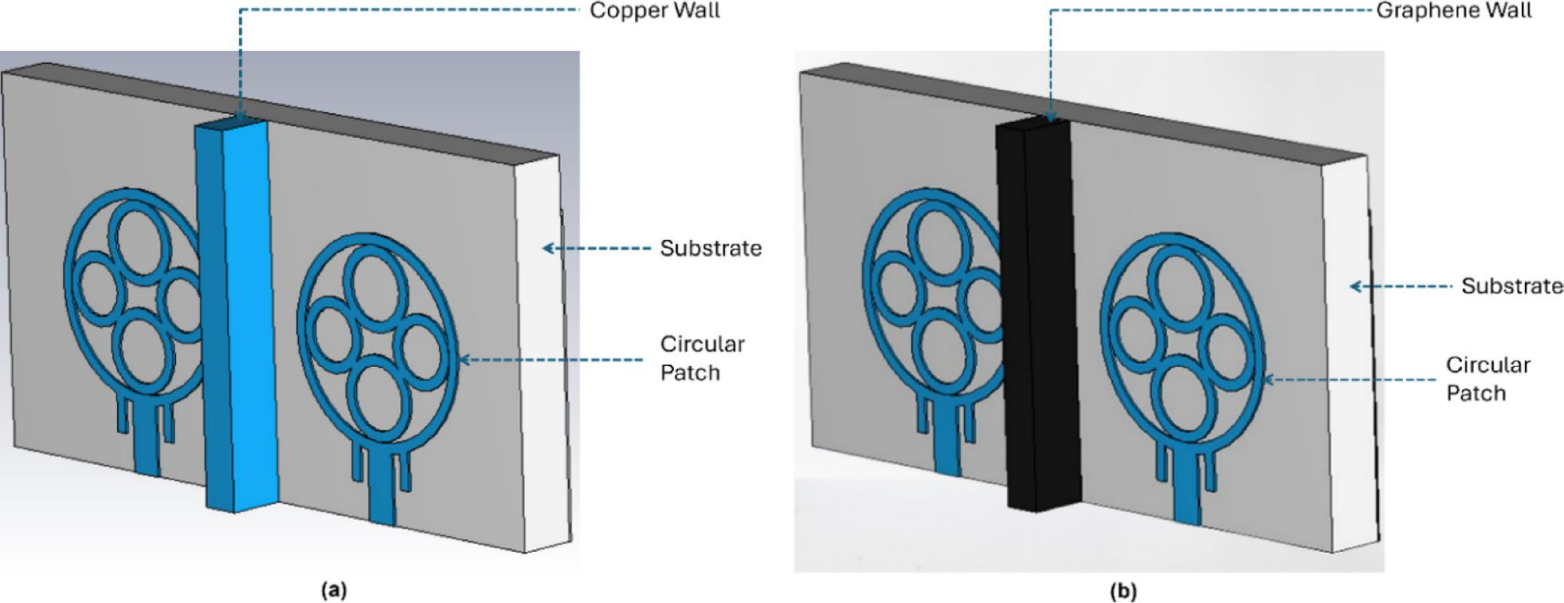
3D Structure of the proposed MIMO antenna with (**a**) Copper and (**b**) Graphene isolation walls.

The electromagnetic independence of individual radiating elements is a critical component in evaluating the performance of MIMO antenna systems, with transmission coefficients (S₂₁, S₁₂) serving as essential metrics to quantify inter-element coupling^35^. Since undesired electromagnetic interactions can severely impair performance by raising signal correlation, decreasing diversity gain, and constricting channel capacity, mutual coupling becomes a crucial design challenge when circular antenna elements are positioned closely together in circular MIMO configurations. As shown in Fig. 7a,b, a comprehensive comparative study was conducted to evaluate three isolation techniques: baseline operation without an isolation barrier, the use of a conventional copper wall, and the incorporation of a graphene-based barrier, leveraging the unique electromagnetic properties of two-dimensional carbon structures^36^.

The superior performance of copper over graphene as an isolation barrier can be attributed to fundamental differences in their electromagnetic properties. Copper possesses a bulk electrical conductivity of approximately 5.96 × 10⁷ S/m, which remains essentially constant across the THz frequency range, enabling highly efficient induced surface currents that generate strong opposing electromagnetic fields, effectively blocking inter-element coupling. In contrast, graphene exhibits a frequency- and bias-dependent complex surface conductivity governed by the Kubo formalism, comprising both intraband and interband contributions. At THz frequencies, graphene’s effective conductivity, although tunable, remains significantly lower than that of copper, resulting in comparatively weaker surface current densities and reduced electromagnetic shielding effectiveness. Furthermore, copper’s well-defined bulk structure supports clearly defined and continuous induced current paths along the barrier surface, minimizing resistive losses and maximizing the reflection of incident electromagnetic fields. Graphene, being an atomically thin two-dimensional material, inherently exhibits higher surface resistance and greater sensitivity to substrate interactions, scattering mechanisms, and material imperfections at THz frequencies, all of which collectively diminish its shielding efficiency relative to copper. Additionally, the dielectric losses associated with graphene’s substrate interface further attenuate the induced surface currents, reducing the barrier’s ability to suppress electromagnetic leakage between adjacent antenna elements.

**Fig. 8:**
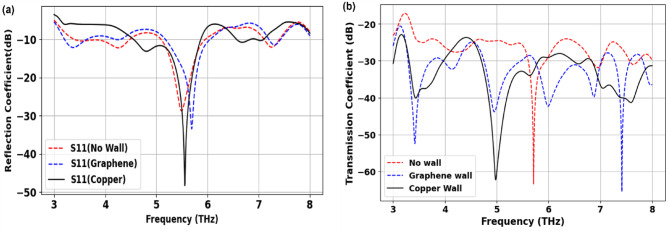
Comparative analysis of (**a**) Reflection and (**b**) Transmission coefficients under various isolation barrier designs.

Figure 8a illustrates the reflection coefficients (S₁₁) under three configurations: without any isolation wall, with a graphene barrier, and with a copper barrier, with the dominant resonance occurring at approximately 5.55 THz in all cases. A progressive improvement in return loss depth is observed with increasing barrier conductivity, consistent with the physical reasoning above. The no-wall configuration yields a minimum return loss of approximately − 28.5 dB, while the graphene barrier improves this to − 33.34 dB due to its partial electromagnetic shielding effect. The copper barrier achieves the deepest resonance of approximately − 48 dB, confirming superior impedance matching attributed to its high conductivity and efficient induced current generation.

Figure 8b presents the transmission coefficients (S₁₂) across the 3–8 THz band, providing a more quantitative distinction between the three configurations. In the absence of any barrier, inter-element coupling remains elevated at approximately − 18 to − 22 dB. The graphene barrier offers moderate isolation improvement of − 21 to − 25 dB, limited by its lower surface conductivity and higher resistive losses at THz frequencies. The copper barrier, benefiting from its superior bulk conductivity, continuous induced current paths, and minimal ohmic losses, delivers the most substantial coupling suppression of − 24 to − 30 dB consistently across the operating band. These results conclusively justify the adoption of the copper barrier as the preferred isolation structure, owing to its physically superior electromagnetic shielding mechanism, thereby ensuring low envelope correlation coefficient (ECC), enhanced port isolation, and optimized MIMO diversity performance for 6G THz communication systems.

### Optimized orientation for best circular MIMO antenna

**Fig. 9:**
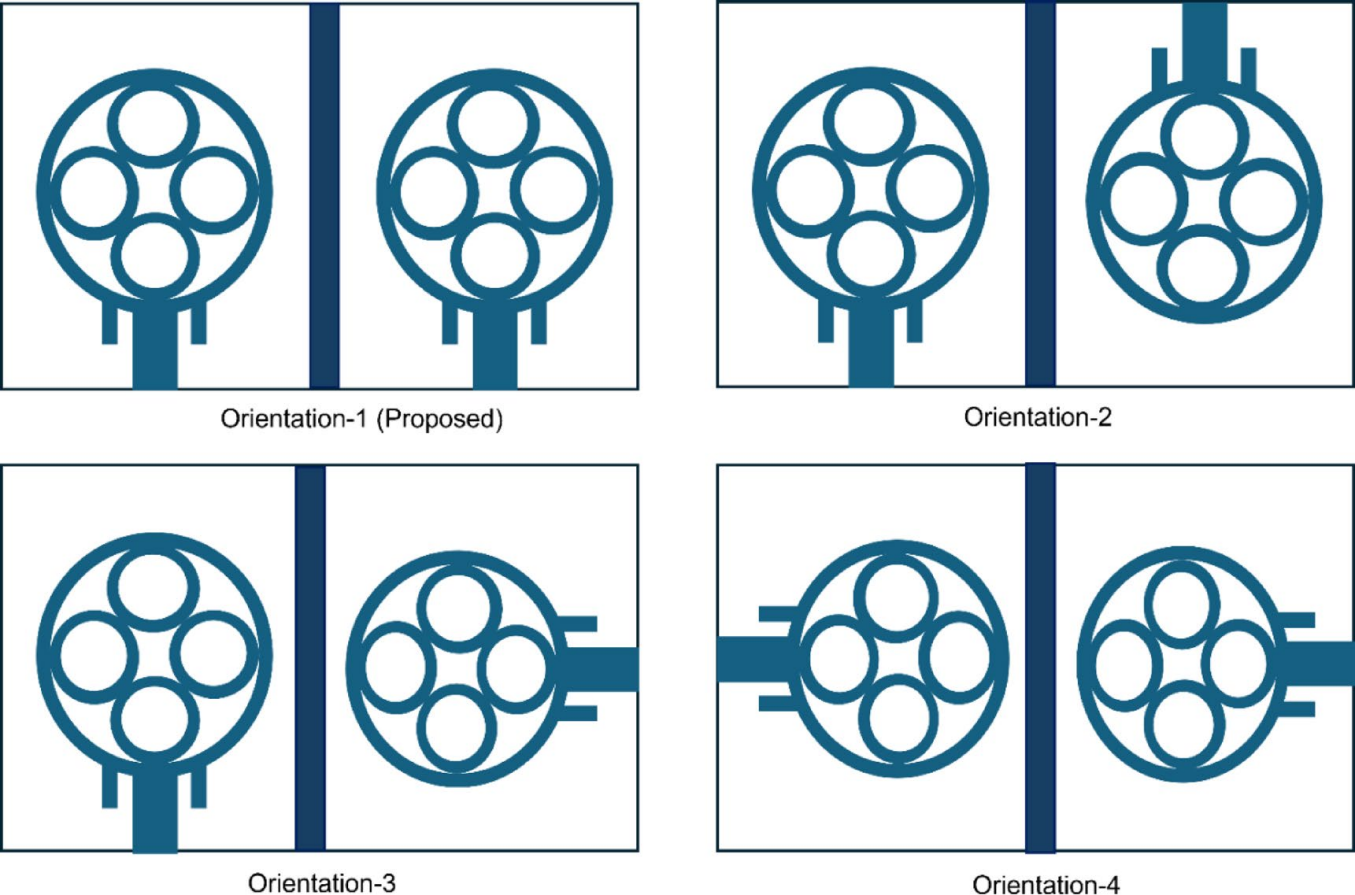
Performance evaluation of four MIMO antenna designs for 6G THz applications.

To achieve an optimized circular MIMO antenna configuration with enhanced electromagnetic isolation, four distinct spatial orientation schemes were systematically investigated through comprehensive parametric analysis, as illustrated in Fig. 9. Configuration 1 (Orientation-1) establishes the baseline arrangement with both antenna elements oriented in identical directions while maintaining optimal spatial separation for reference performance evaluation. Configuration 2 (Orientation-2) implements a 180° rotational transformation of the second element relative to the first, creating an opposite-facing configuration that exploits pattern diversity to reduce mutual coupling. Configuration 3 (Orientation-3) employs a 90° angular displacement, positioning the antenna elements in perpendicular alignment to maximize polarization diversity and minimize electromagnetic field overlap.

**Fig. 10:**
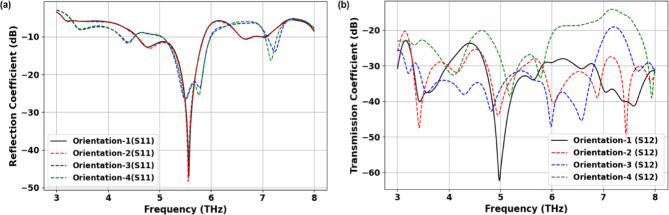
Performance comparison of circular MIMO antenna: (**a**) Reflection (S₁₁) and (**b**) Transmission Coefficients (S₁₂).

Configuration 4 (Orientation-4) arranges the elements in a side-by-side symmetric configuration with 360° rotational consideration, establishing a balanced lateral layout that optimizes current distribution symmetry. This comparative evaluation identifies the optimal spatial arrangement that maximizes inter-element isolation while preserving the radiation characteristics and bandwidth of the circular patches, thereby ensuring enhanced circular MIMO system performance through geometric optimization. Figure 10 comparatively evaluates the electromagnetic performance of four distinct antenna orientations. Figure 10a presents the reflection coefficient (S₁₁), whereas Fig. 10b illustrates the transmission coefficients (S₁₂), which quantify the mutual coupling between antenna elements. As observed from Fig. 10a, all four configurations exhibit a well-defined single-band resonance centered at 5.55 THz, confirming consistent impedance matching characteristics across the proposed orientations. The minimum return loss for Orientations 3 and 4 is approximately − 25 dB, indicating acceptable impedance matching at the resonant frequency. However, a clear distinction emerges in terms of isolation performance. From Fig. 10b, Orientation 1 demonstrates strong mutual coupling suppression, maintaining an isolation level of approximately − 23 dB. Orientation 2 also resonates at 5.55 THz and achieves a comparable isolation level of around − 20 dB. In contrast, Orientation 3 exhibits the highest isolation performance, reaching approximately − 25 dB, while Orientation 4 achieves an isolation level of about − 23 dB. Overall, although Orientations 2 and 3 provide higher isolation compared to Orientation 1, the reflection coefficient (S₁₁) of Orientation 1 is significantly better than that of Orientations 2 and 3, making it the most balanced configuration in terms of both impedance matching and mutual coupling suppression.

## Comparative analysis of single-element and circular MIMO antenna

### Bandwidth evaluation in single-element and circular MIMO antenna

**Fig. 11:**
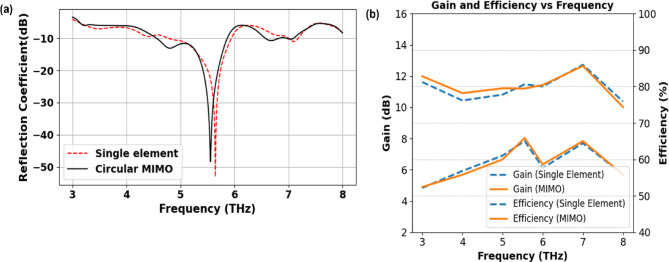
Comparative analysis of (**a**) Reflection Coefficient, (**b**) Gain, and efficiency in single and MIMO Antennas.

The reflection characteristics shown in Fig. 11a, evaluated in terms of the magnitude of the reflection coefficient ∣S_11_∣, demonstrate a significant bandwidth enhancement achieved through the implementation of the MIMO configuration^37^. The single-element circular patch antenna exhibits an operational bandwidth of 1.10 THz (4.83–5.93 THz), satisfying the ∣S_11_∣<−10 dB criterion for acceptable impedance matching. In comparison, the proposed MIMO antenna provides an expanded bandwidth of 1.30 THz (4.56–5.86 THz), corresponding to an 18% improvement over the single-element design. This enhancement is primarily attributed to electromagnetic coupling and mutual interactions between the radiating elements in the MIMO array, which modify the overall input impedance and introduce additional resonant modes, thereby extending the effective operating frequency range.

### Efficiency analysis of single-element versus circular MIMO antenna

As illustrated in Fig. 11b, the efficiency analysis indicates that the single-element circular patch antenna achieved a radiation efficiency of 85.96%, while the circular MIMO configuration attained 85.64%, reflecting a minimal reduction of only 0.32%. These efficiency values were obtained through full-wave simulations in both CST and HFSS, where all dominant loss mechanisms were rigorously accounted for. Specifically, conductor losses in the graphene radiating elements were modeled using a frequency-dependent complex surface conductivity based on the Kubo formalism, which captures both intra band and inter band contributions at THz frequencies. Dielectric losses in the substrate were incorporated through the frequency-dependent complex permittivity, with the loss tangent (tan δ) applied across the entire 3–8 THz simulation band. Surface wave losses, which are particularly significant at THz frequencies, were inherently captured through the full-wave boundary condition setup, including perfectly matched layer (PML) absorbing boundaries, ensuring accurate accounting of power leakage into substrate modes. The slight efficiency degradation from single-element to MIMO configuration (only 0.32%) is primarily attributed to mutual coupling effects and increased feeding network complexity. Despite this, the circular MIMO configuration provides substantial system-level advantages, including spatial diversity, enhanced channel capacity, and beamforming capability. The negligible efficiency difference (less than 0.4%) confirms that both designs exhibit excellent radiation performance, making the circular MIMO antenna well-suited for advanced high-data-rate platforms such as 6G THz communication networks, where diversity gain and spectral efficiency are critical.

### Comparative gain analysis of single-element and circular MIMO

The antenna gain analysis indicates that the single-element circular patch achieves a peak gain of 7.83 dB at 5.55 THz, demonstrating excellent performance for a compact single-radiator structure operating at terahertz frequencies. In comparison, the circular MIMO antenna exhibits an enhanced gain of 8.038 dB at the same frequency, corresponding to a 0.208 dB improvement. This gain increment results from constructive interference generated by the coherent excitation of multiple radiating elements within the MIMO configuration. As illustrated in Fig. 11b, this enhancement, although modest in magnitude, is significant for advanced THz communication systems where even fractional decibel improvements contribute to increased link reliability, improved signal-to-noise ratio, and extended transmission range. Therefore, while the single-element design offers strong standalone performance, the circular MIMO configuration provides superior system-level gain characteristics, making it more suitable for next-generation high-capacity THz communication networks.

## Results and analysis of circular MIMO antenna design

### Evaluation of reflection and transmission coefficients

**Fig. 12:**
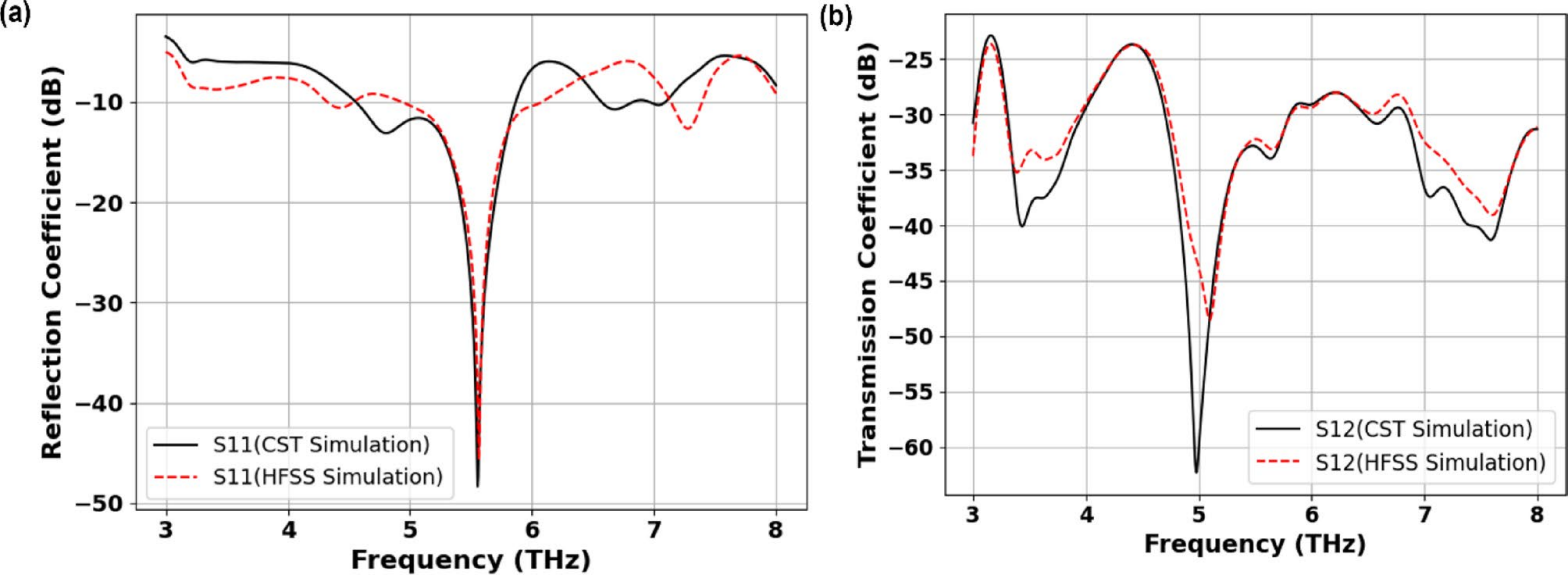
Comparison of CST and HFSS simulations for (**a**) Reflection coefficient and (**b**) Mutual coupling.

The performance of the proposed circular MIMO antenna is comprehensively evaluated through both CST and HFSS simulations, demonstrating strong overall agreement between platforms and validating the design’s robustness^38^. Figure 12a presents the return loss (|S11|) characteristics over the 3–8 THz range, where a deep resonance dip of approximately − 47 dB occurs at 5.55 THz, indicating excellent impedance matching at the operating frequency.

At this resonant point, the |S11| value falls well below the − 10 dB threshold, confirming efficient power transfer, minimal reflection, and strong resonant performance at the desired THz frequency. Figure 12b illustrates the transmission coefficient (S_12_), where both simulation platforms consistently achieve isolation levels of approximately − 23 dB, confirming effective suppression of mutual coupling between the antenna elements. Although a minor discrepancy is observable between the CST and HFSS results across certain frequency sub-bands, this deviation is physically attributable to the fundamental differences in the numerical solvers employed by each platform. CST utilizes the Finite Integration Technique (FIT) with time-domain solving, whereas HFSS employs the Finite Element Method (FEM) in the frequency domain. These methodological differences lead to slight variations in mesh generation, boundary condition enforcement, and material model interpolation, particularly at THz frequencies where geometrical features are electrically large and substrate dispersion effects become more pronounced. Furthermore, minor discrepancies in the convergence criteria and port excitation definitions between the two tools can introduce small but visible offsets in the simulated S-parameter responses. Despite these differences, both solvers predict the same resonant frequency, the same deep null in S_11_, and comparable isolation levels in S_12_, confirming that the observed deviations are solver-intrinsic rather than design-related. The overall consistency between CST and HFSS validates the computational reliability and design accuracy of the proposed structure, confirming its suitability for high-performance THz MIMO communication systems.

### Envelop correlation coefficient (ECC) and directivity gain (DG)

The correlation between signal envelopes received at various antenna elements is measured by the Envelope Correlation Coefficient (ECC), which is a crucial parameter for assessing MIMO antenna performance^39^. ECC values close to 0 indicate uncorrelated signals, while values near 1 reflect strong correlation. For MIMO systems, low ECC (ideally < 0.5) is essential to ensure spatial multiplexing, diversity gain, and higher data rates. High ECC degrades interference suppression, channel capacity, and spectral efficiency. Achieving low ECC requires careful design with proper antenna spacing, orthogonal radiation patterns, polarization diversity, and suitable propagation conditions. The ECC is calculated from the S-parameters and is mathematically expressed in Eq. (3).

3 $$\:ECC=\frac{{{|S}_{11}^{*}{S}_{12}-{S}_{21}^{*}{S}_{22}|}^{2}}{(1-{\left|{S}_{11}\right|}^{2}-{\left|{S}_{21}\right|}^{2})(1-{\left|{S}_{22}\right|}^{2}-{\left|{S}_{12}\right|}^{2})}$$

**Fig. 13:**
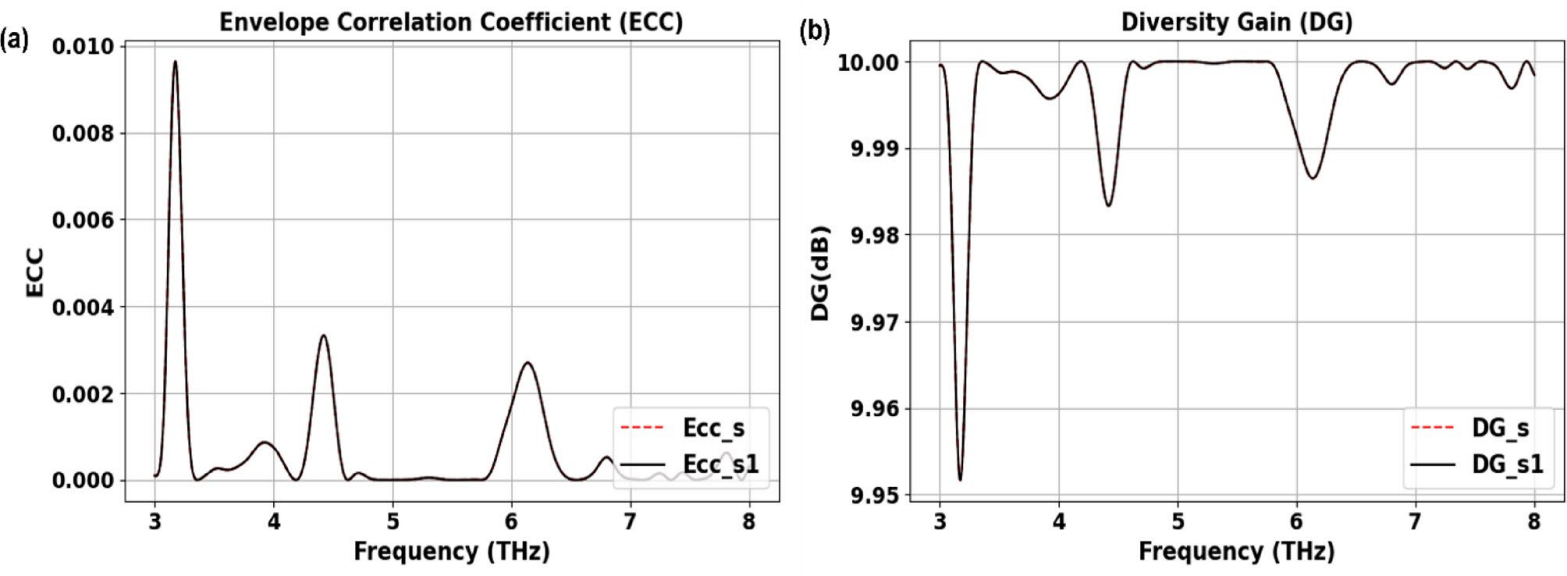
Performance of (**a**) ECC and (**b**) DG in the proposed circular MIMO antenna.

The Envelope Correlation Coefficient (ECC) of the proposed 2-port circular MIMO antenna, shown in Fig. 13a, remains extremely low across the entire operating band, varying from approximately 0.0035 to nearly zero. The two evaluated curves (ECC_s derived from S-parameters and ECC_s1 obtained from far-field radiation patterns) closely overlap, demonstrating strong consistency between the calculation methods. Such near-zero ECC values indicate excellent port isolation and minimal signal correlation between antenna elements, ensuring strong spatial diversity and efficient spatial multiplexing. These outstanding ECC characteristics confirm the suitability of the proposed circular MIMO antenna for advanced 6G terahertz (THz) communication systems, where high channel capacity and reliable diversity performance are essential.

Diversity Gain (DG) quantifies the improvement in signal quality, system reliability, and signal-to-noise ratio achieved through antenna diversity techniques^40^. The DG performance shown in Fig. 13b presents two closely overlapping curves: DG_s, calculated using S-parameter-based envelope correlation coefficient (ECC) values, and DG_s1, derived from far-field radiation characteristics. While DG_s evaluates diversity performance based on network parameters, DG_s1 provides a radiation-based assessment, offering a more comprehensive field-oriented perspective. The Diversity Gain is defined in Eq. (4).

4 $$\:DG=10\sqrt{1-{ECC}^{2}}$$

The proposed circular MIMO antenna achieves a diversity gain of 9.985 to nearly 10 across the operating band, with DG_s and DG_s1 showing negligible differences, thereby confirming strong agreement between the two evaluation methods. The proposed circular MIMO antenna achieves an outstanding diversity gain ranging from 9.985 to nearly 10 across the operating frequency band, closely approaching the theoretical maximum value of 10 (Fig. 13b). Superior signal dependability and low correlation are demonstrated by this exceptional performance, demonstrating its good applicability for THz circular MIMO applications and validating its potential for high-performance 6G communication systems.

### CCL, TARC, MEG, and multiplexing efficiency

#### Channel capacity loss (CCL)

One important indicator for MIMO performance is Channel Capacity Loss (CCL), which measures the throughput drop from the theoretical maximum brought on by inter-antenna correlation. While ideal MIMO systems scale their capacity linearly with more antennas, channel correlation problems cause degradation in real-world implementations. CCL offers crucial insight into these performance constraints, facilitating precise evaluation of theoretical potential against actual system capacity. CCL is represented by Eq. (5)^41^.

5 $$\:CCL={-\mathrm{log}}_{2}\mathrm{d}\mathrm{e}\mathrm{t}\left({p}_{R}\right)$$

where Eq. (6) is used to represent $\:{P}_{R}$, the receiving antenna’s correlation matrix.

6 $$\:{p}_{R}=\left[\begin{array}{cc}{\rho\:}_{xx}&\:{\rho\:}_{xy}\\\:{\rho\:}_{yx}&\:{\rho\:}_{yy}\end{array}\right]$$

Where $$\:{\rho\:}_{xx}=1-{(\left|{S}_{xx}\right|}^{2}+{\left|{S}_{xy}\right|}^{2})$$, $$\:{\rho\:}_{yy}=1-{(\left|{S}_{yy}\right|}^{2}+{\left|{S}_{yx}\right|}^{2})$$, $$\:{\rho\:}_{xy}={{|S}_{xx}^{*}{S}_{xy}+{S}_{yx}^{*}{S}_{xy}|}^{2}$$, $$\:{\rho\:}_{yx}={{|S}_{yy}^{*}{S}_{yx}+{S}_{xy}^{*}{S}_{yx}|}^{2}$$.

Whereas $\:{\left|{S}_{xy}\right|}^{2}$ and $\:{\left|{S}_{yx}\right|}^{2}$ show the power connected to the opposite port, signifying mutual coupling between the antennas, $\:{\left|{S}_{xx}\right|}^{2}$ and $\:{\left|{S}_{yy}\right|}^{2}$ show the reflected power at ports X and Y, respectively.

**Fig. 14:**
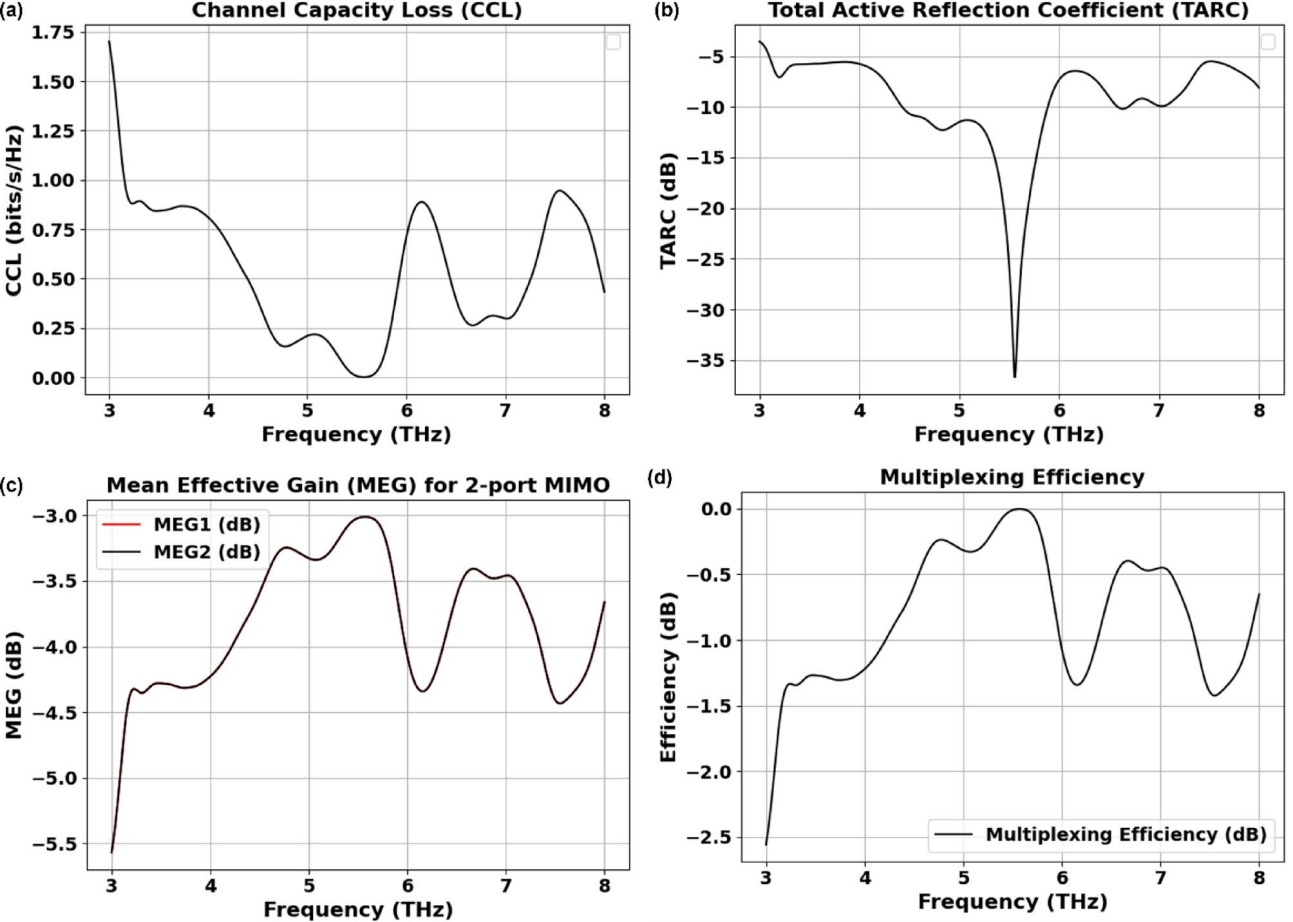
Performance of the proposed circular MIMO antenna: (**a**) CCL and (**b**) TARC (**c**) MEG (**d**) multiplexing efficiency.

The channel capacity loss (CCL) as a function of frequency over the 3–8 THz range is shown in Fig. 14a. Lower values of CCL indicate less inter-element correlation and more data transmission capacity^42^. CCL measures the decrease in channel capacity brought on by correlation between antenna elements. The results indicate that the 4.56–5.86 THz operating bandwidth exhibits the lowest channel capacity loss (CCL), ranging from 0.25 bits/s/Hz to nearly zero. This minimal CCL reflects optimal antenna performance and maximum achievable channel capacity within this frequency band. Therefore, this range is particularly well-suited for high-capacity THz communication systems.

#### Total active reflection coefficient (TARC)

A crucial performance indicator for MIMO antenna systems, the TARC assesses the overall reflection behavior when several antenna elements are operating concurrently^2^. A more accurate indicator of system performance, TARC takes into account the mutual interactions between all elements under concurrent excitation with different phases and amplitudes, in contrast to traditional reflection coefficients that evaluate individual elements separately^43^. It is expressed by Eq. (7)

7 $$\:TARC=\frac{\sqrt{{(\left|{S}_{xx}\right|+\left|{S}_{xy}\right|)}^{2}+{(\left|{S}_{yy}\right|+\left|{S}_{yx}\right|)}^{2})}}{\sqrt{2}}$$

In MIMO systems, a lower TARC value denotes better impedance matching and less power reflection, which immediately translates to increased performance and radiation efficiency. Although practical MIMO applications typically allow TARC values below − 10 dB (ensuring less than 10% input power reflection), Fig. 14b shows remarkable performance, with TARC falling below − 35 dB at roughly 5.55 THz. This indicates excellent impedance matching and low reflection losses that maximize radiated power while minimizing inter-element interference at this ideal frequency.

#### Mean effective gain (MEG)

The average power received by each antenna element in realistic propagation situations is measured by Mean Effective Gain (MEG), a crucial statistic in MIMO antenna systems^44^. In contrast to traditional free-space gain, MEG takes into consideration polarization diversity, multipath, and angular power distribution. It is commonly represented in dB and is the ratio of the total incoming power from all directions and polarizations to the mean received power at an element. Using the S-parameters, the MEG of port *i* for a 2-port MIMO antenna system is determined using Eq. (8).

8 $$MEG_{i} = \frac{1}{2}\left( {1 - \mathop \sum \limits_{{j = 1}}^{2} \left| {S_{{ij}} } \right|^{2} } \right)$$

Here, Power transferred from port j to port i is represented by the S-parameter $\:{S}_{ij}$ in this case.

The MEG performance of the proposed 2-port circular MIMO antenna over the 3–8 THz range is illustrated in Fig. 14c. For optimal MIMO operation under uniform isotropic conditions, the MEG of each port should ideally be close to − 3 dB. Within the operating bandwidth (4.56–5.86 THz), both MEG1 and MEG2 vary between approximately − 3.5 dB and − 3 dB, exhibiting nearly identical trends with negligible deviation between the two ports. This close agreement confirms balanced power distribution and symmetrical radiation behavior, indicating low mutual coupling and consistent radiation efficiency. Furthermore, the stable MEG response supports the high multiplexing efficiency observed in Fig. 14d, reinforcing the antenna’s capability for reliable diversity performance and robust 6G THz communication applications.

#### Multiplexing efficiency

A crucial performance statistic in MIMO antenna systems, multiplexing efficiency (ME) measures how well independent data streams can be transmitted and received simultaneously across several antenna elements^45^. ME comprehensively accounts for both individual antenna radiation efficiency and inter-element correlation effects, providing a unified measure of the system’s spatial multiplexing capabilities. The mathematical formulation of ME is expressed in Eq. (9), which establishes the relationship between these key parameters to determine overall MIMO system performance.

9 $${\mathrm{Multiplexing}}\;{\mathrm{Efficiency}}\left( {{\mathrm{dB}}} \right) = {\mathrm{1}}0log_{{10}} [({\mathrm{1}} - \left| \rho \right|^{2} )({\mathrm{1}} - \left| {S_{{11}} } \right|^{2} - \left| {S_{{21}} } \right|^{2} )]$$

Here, ρ is the ECC, (1-$\:{\left|\rho\:\right|}^{2}$) is the correlation efficiency and (1-$\:{\left|{S}_{11}\right|}^{2}$-$\:{\left|{S}_{21}\right|}^{2}$) is total radiation efficiency of port 1. Figure 14d illustrates the multiplexing efficiency of the proposed circular MIMO antenna system over the 3–8 THz frequency range. Within the operating bandwidth of 4.56–5.86 THz, the system maintains consistently high efficiency, ranging from − 0.5 dB to nearly 0 dB. These excellent results confirm the antenna’s capability to achieve near-maximum channel capacity and strong diversity performance, thereby demonstrating its suitability for high-throughput 6G THz MIMO communication systems.

### Current distribution

**Fig. 15:**
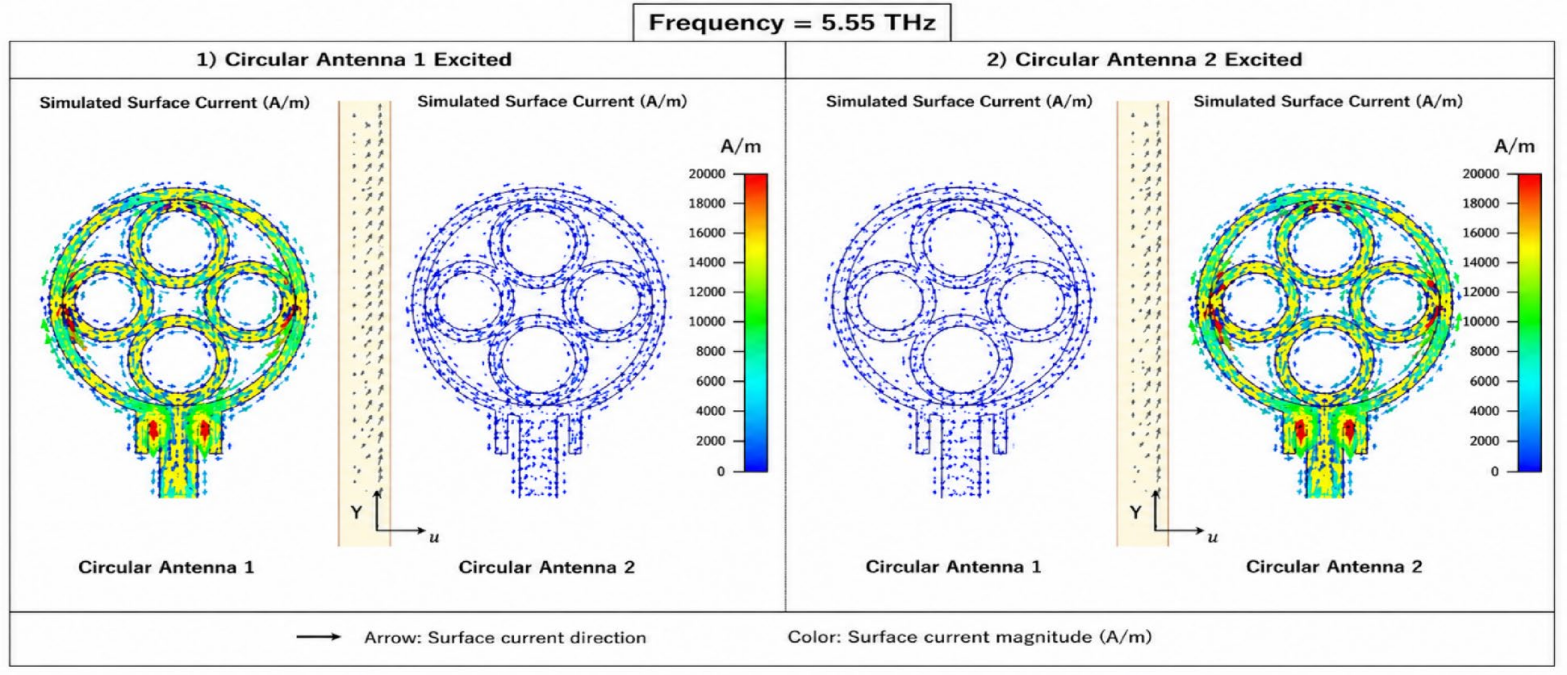
Current Distribution at 5.55 THz: (**a**) Port 1 Off/Port 2 On, (**b**) Port 1 On/Port 2 Off.

Figure 15 illustrates the simulated surface current distribution at 5.55 THz for the proposed circular MIMO antenna comprising four inner circular elements. The color scale ranges from blue (low current density, approximately 2000 A/m) to red (high current density, approximately 20,000 A/m), while directional arrows indicate the surface current flow pattern. In case 16(a), when Circular Antenna 1 is excited, strong surface current concentrations are observed along the outer ring and interconnecting regions, with pronounced hotspots reaching peak magnitudes of approximately 20,000 A/m near the junctions between the outer boundary and the inner circular elements. Critically, Circular Antenna 2 exhibits substantially suppressed induced currents, remaining at considerably lower magnitude levels close to 2000 A/m, representing nearly a tenfold reduction in surface current density. This quantitative contrast directly evidences strong electromagnetic decoupling between the two antenna ports and supports the high isolation performance reflected in the S-parameter results. In case 15(b), when Circular Antenna 2 is excited, the current distribution shifts significantly, with hotspots relocating predominantly to the lower feed region and adjacent circular elements at comparable peak magnitudes of approximately 20,000 A/m, while Circular Antenna 1 exhibits only weak residual current responses near 2000 A/m. The directional arrow overlays further confirm that current flow remains well-confined within the excited antenna structure, with negligible coupling propagating toward the inactive port. This port-selective excitation behavior, supported by quantitative surface current magnitude comparisons across both cases, demonstrates the design’s capability to achieve spatially distinct and controlled electromagnetic field distributions at 5.55 THz, directly linking the observed current confinement to the strong inter-port isolation performance of the proposed circular MIMO antenna.

### Radiation pattern

**Fig. 16:**
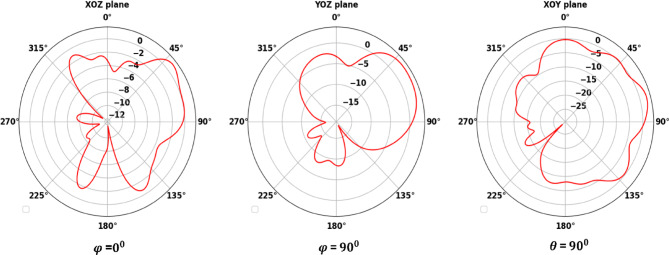
Radiation characteristics of the proposed circular MIMO antenna (**a**) φ=$\:{0}^{0}$ (**b**) φ=$\:{90}^{0}$ (**c**) θ = $\:{90}^{0}$at 5.55 THz.

The radiation pattern characterizes the spatial distribution of electromagnetic power radiated by the antenna and is a key parameter for assessing directivity, coverage, and overall performance^46^. The observed radiation characteristics are governed by the combined effects of the circular geometry, slot configurations, and optimized ground plane design. The radiation characteristics observed in Fig. 16 are primarily influenced by the combined effects of the circular antenna geometry, slot configurations, and the optimized ground-plane structure. At φ = 0° (E-plane), the radiation pattern exhibits a quasi-directional behavior with a dominant main lobe directed toward the broadside (approximately 45°–90°). This is primarily due to the circular patch geometry, which supports concentrated surface current distribution along its perimeter, thereby reinforcing radiation in the forward direction. The presence of slots introduces controlled perturbations in the current paths, which suppress unwanted higher-order modes and reduce side-lobe levels, resulting in a stable forward-directed radiation pattern with limited back radiation. At φ = 90° (H-plane), the pattern becomes smoother and more symmetric with a wider main lobe. This behavior arises from the inherent rotational symmetry of the circular structure, which promotes uniform current distribution along the antenna edges. The reduced side-lobe levels and the null around 180° are attributed to the optimized ground plane, which acts as a reflective surface that suppresses backward radiation and enhances the front-to-back ratio. At θ = 90° (azimuthal plane), the radiation pattern is nearly omnidirectional with minor variations in amplitude. This is a direct consequence of the circular geometry, which enables azimuthally symmetric current flow and uniform field distribution in the horizontal plane. The slot configurations introduce slight controlled asymmetries that stabilize the pattern without significantly affecting omnidirectional coverage, which is particularly beneficial for MIMO systems requiring spatial diversity. Overall, the quasi-directional E-plane behavior, symmetric H-plane response, and near-omnidirectional azimuthal pattern are directly explained by the circular radiating geometry, slot-induced current shaping, and ground plane optimization, confirming the stable radiation performance of the proposed antenna at 5.55 THz (Fig. 16).

## Machine learning analysis

**Fig. 17:**
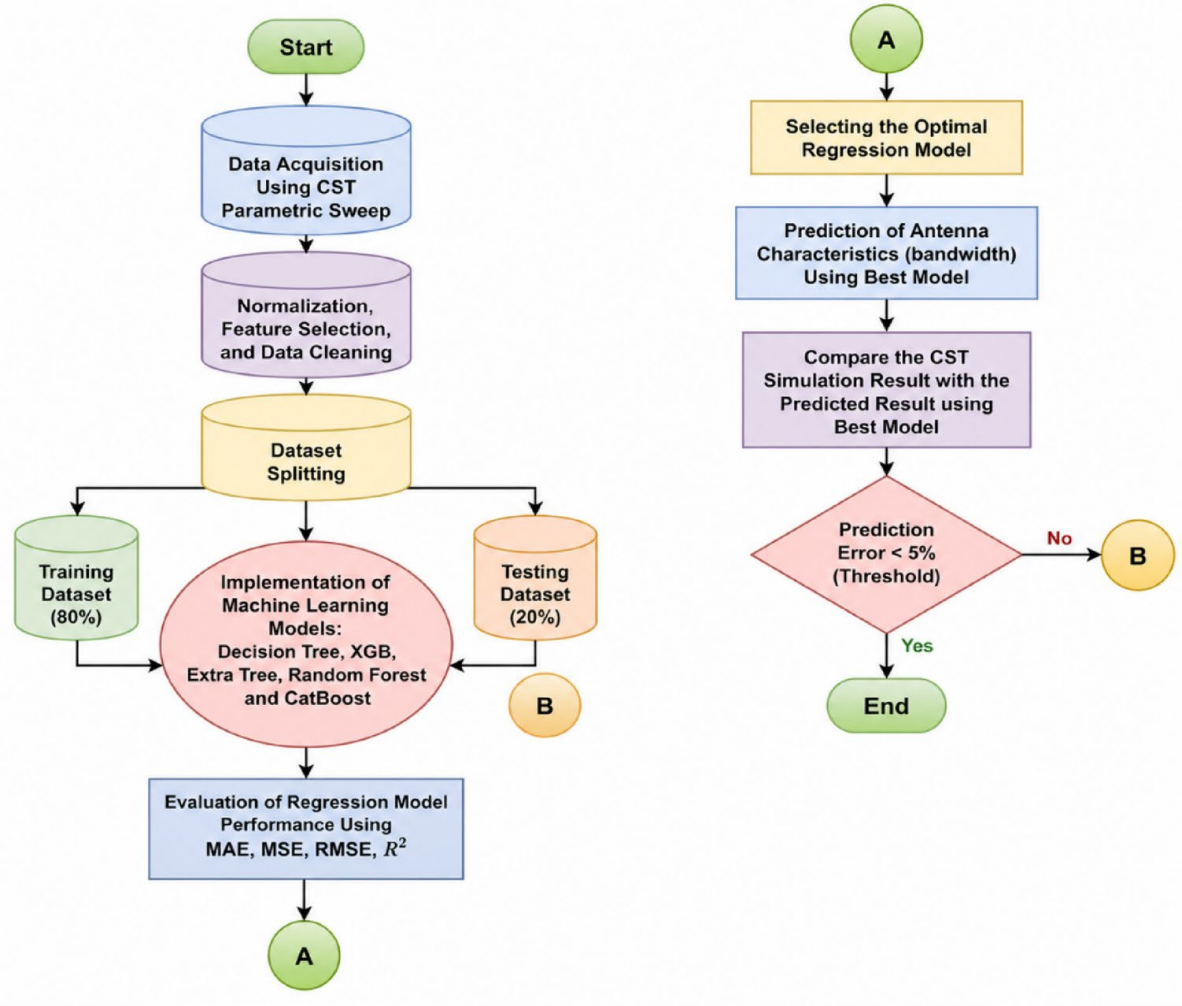
ML-Assisted performance assessment of the proposed circular MIMO antenna.

Commercial electromagnetic simulation tools such as CST and HFSS impose significant computational burdens on antenna design, requiring long processing times and complex optimization algorithms to achieve desired performance. These challenges intensify in compact MIMO systems, where multiple radiating elements on shared substrates and ground planes demand careful management of inter-element coupling, spatial limitations, and simultaneous multi-port optimization^47^. To overcome these constraints, researchers increasingly employ ML regression models that can rapidly predict antenna dimensions or performance metrics from geometrical parameters, effectively replacing iterative simulations with predictive modeling. By systematically exploring large configuration spaces, ML methods identify optimal parameter sets for key MIMO performance indicators such as gain, bandwidth, isolation, and radiation patterns while incorporating mutual coupling and spatial diversity effects. This data-driven approach accelerates design iterations, reduces computational costs, and minimizes reliance on physical prototyping, ultimately enabling faster and more efficient MIMO antenna development. Figure 17 illustrates a comprehensive machine-learning workflow systematically developed for predicting the bandwidth and isolation performance of a circular MIMO antenna through a structured and data-driven approach. The methodology begins with data acquisition obtained from CST-based parametric sweeps, generating a large and diverse dataset that captures the influence of key geometrical parameters on antenna behavior. The collected data then undergo essential preprocessing steps, including data cleaning, normalization, and feature selection, to construct a refined and optimized input dataset suitable for ML applications. The processed dataset is subsequently divided into training and testing subsets using an 80:20 ratio, ensuring reliable and unbiased model evaluation. Multiple supervised regression algorithms, including Decision Tree, XGBoost, Extra Tree, Random Forest, and CatBoost, are implemented and trained using the training dataset to enable comprehensive comparative analysis. Model performance is quantitatively assessed using standard evaluation metrics such as MAE, MSE, RMSE, and R². Based on these metrics, the optimal regression model is selected and employed to predict antenna characteristics, specifically bandwidth and isolation. The predicted results are then compared with CST simulation outputs, and a prediction error threshold of 5% is used as an acceptance criterion. This systematic workflow ultimately establishes an efficient and reliable ML-based predictive framework for accurate and rapid circular MIMO antenna performance estimation.

### Analysis of dataset generation and overview

To develop and validate the ML models for the proposed circular MIMO antenna, a structured dataset of 240 samples with 14 features was systematically generated through full-wave parametric simulations in CST and HFSS using a Design of Experiments (DoE) approach. Each geometric parameter was varied independently within its practical design range while maintaining identical boundary conditions, material definitions, and excitation settings across all simulation runs, ensuring dataset consistency and eliminating solver-induced variance.

**Table 2:** Statistics of dataset structure for 2-port circular MIMO antenna.

Crucial features	Parameter	Explanation
Size of dataset	240 samples $\:\times\:$ 14 features	240 different rows of combinations with 14 different output-input parameters.
Splitting dataset	Training dataset = 192 (80%), Testing dataset = 48 (20%)	Training data are used to develop ML models, while testing data validate prediction performance.
Geometric features (Inputs)	Ten inputs=$\:{W}_{f}$, R_2_, R_3_, R_4_, R_5_, Sl, L_g_, t, h, d	Feedline widths, Outer and Inner radius of small circles, stub length, ground length, thickness of the patch, height of substrate and distance between circular patches.
Performance metrics (Outputs)	Three outputs = F, BW, Isolation	Frequency, Bandwidth and Isolation.

Table 2 presents the overall structure of the dataset developed for the 2-port circular MIMO antenna. It defines both the input feature space and the corresponding output responses used for machine learning modeling. The input dataset consists of ten key geometrical parameters, including feedline width (W_f_), radii of multiple circular elements (R_2_, R_3_, R_4_, and R_5_), stub length (Sl), ground length (Lg), substrate height (h), metal thickness (t), and inter-element spacing (d). These parameters are systematically selected and varied within realistic design constraints to comprehensively capture their influence on the antenna’s electromagnetic behavior. The output variables include resonant frequency (F), bandwidth (BW), and isolation, which serve as critical performance indicators for evaluating the effectiveness of the proposed THz MIMO antenna design. Table 3 provides a detailed description of the dataset generation range and discretization strategy for the 2-element circular MIMO antenna. It specifies the variation limits and step sizes for each geometrical parameter used during data generation. The feedline width is varied from 2 to 6 μm with a step of 0.4 μm, while different circular radii parameters (R_2_, R_3_, R_4_, and R_5_) are varied within defined ranges using fine step increments to capture subtle geometric effects on antenna performance. The substrate height (h), metal thickness (t), center-to-center spacing between antenna elements, ground length (Lg), and stub length (Sl) are also systematically varied within practical fabrication constraints. Notably, some parameters such as ground length and element spacing are varied with relatively larger step sizes to reduce computational cost while maintaining design coverage. Overall, this structured parameter sweep ensures a well-distributed dataset that effectively captures the complex relationship between geometry and electromagnetic response, enabling accurate and reliable machine learning-based prediction of antenna performance.

**Table 3:** Dataset of 2-element circular MIMO antenna.

Feature	Range (µm)	Step size
Feedline width ($\:{W}_{f})$	2–6	0.4
Outer radius of small circle 1,3 (R_2_)	7–9	0.4
Inner radius of small circle 1,3 (R_4_)	7–8	0.2
Outer radius of small circle 2,4 (R_3_)	6–7	0.2
Inner radius of small circle 2,4 (R_5_)	5–7	0.2
Inner radius of radiator 2 (R_5_)	3–5	0.5
Inner radius of radiator 1 (R_3_)	3–5	0.5
Substrate height (h)	9–13	1
Metal Thickness (t)	0.1–0.5	0.1
Center to center distance between 2 element circular antennas	55–80	5
Length of ground (L_g_)	75 − 60	5
Stub length (Sl)	6.5–8.5	0.5

The dataset generation process ensures sufficient diversity and comprehensive coverage of the design space, enabling robust model training. Hyperparameter optimization was performed using grid search combined with 5-fold cross-validation on the training set to achieve an optimal balance between bias and variance, thereby improving prediction accuracy and generalization capability. Additionally, feature importance analysis was conducted to quantify the contribution of each geometric parameter to the predicted outputs, resonant frequency (F), bandwidth (BW), and isolation, enhancing model interpretability and identifying the dominant design factors influencing antenna performance while reducing overfitting risk. Although larger datasets are generally desirable, the use of 240 samples represents a practical compromise considering the high computational cost of full-wave simulations and remains consistent with similar studies. The DoE-based sampling strategy ensures effective design space exploration, and cross-validation further confirms the reliability and generalization capability of the proposed model, with future work aimed at expanding the dataset for even greater accuracy.

### Histogram analysis of dataset parameters

**Fig. 18:**
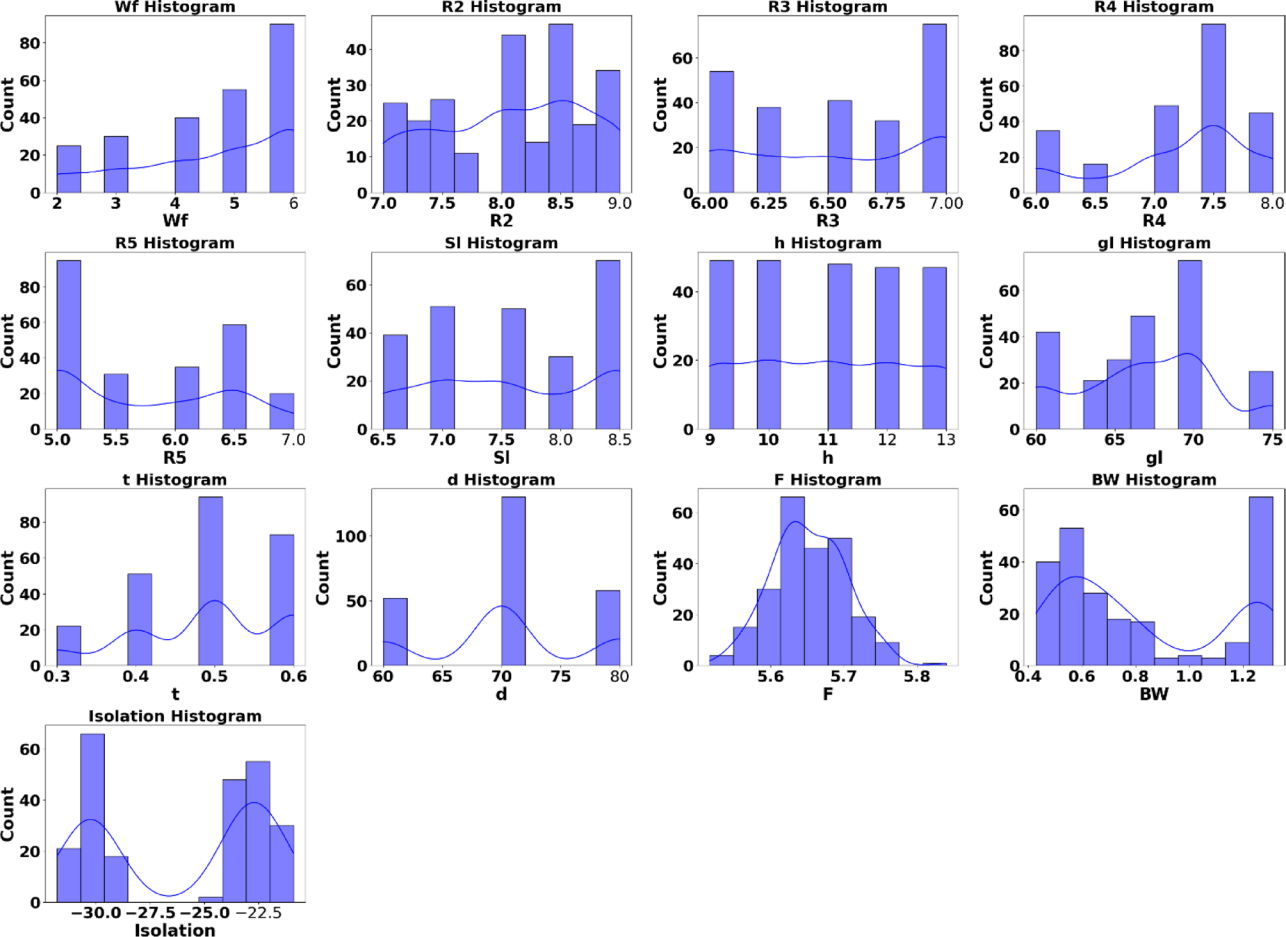
Characterization of design parameters and performance metrics in the proposed dataset.

Histograms are used to visualize the statistical distribution of each parameter across the generated samples, enabling verification of adequate design space exploration, identification of any clustering tendencies, and assessment of output variability for reliable machine learning (ML) model development. Figure 18 presents the histogram distributions of all ten geometric input parameters and three output performance metrics for the 240-sample dataset, along with kernel density estimation (KDE) curves to highlight overall distribution trends. Among the input parameters, the feedline width (W_f_) shows a right-skewed distribution concentrated toward higher values (5–6 μm), while R_2_ and R_4_ exhibit relatively uniform distributions, indicating good coverage of the design space. In contrast, R_3_ and R_5_ display multi-peaked patterns, suggesting a non-linear influence on antenna performance. The stub length (Sl) is biased toward larger values, whereas the ground length (gl) demonstrates a broad spread across its range. The patch thickness (t) is mainly concentrated between 0.3 and 0.6 μm, while the substrate height (h) is fairly uniform from 9 to 13 μm. Additionally, the inter-element spacing (d) shows a bimodal distribution, reflecting its strong impact on mutual coupling behavior. For the output parameters, the resonant frequency (F) is tightly distributed between 5.55 and 5.75 THz, centered around the intended operating frequency. The bandwidth (BW) spans from 0.4 to 1.3 THz and is slightly skewed toward lower values. The isolation ranges from − 30 to − 22.5 dB and exhibits a bimodal distribution, indicating the presence of distinct coupling regimes within the dataset. Overall, these histogram distributions confirm sufficient diversity in both input and output parameters, ensuring that the dataset is well-balanced and suitable for training accurate, robust, and generalizable ML models for THz MIMO antenna performance prediction.

### Evaluating and Selecting Regression Models for Predictive Analysis

**Fig. 19:**
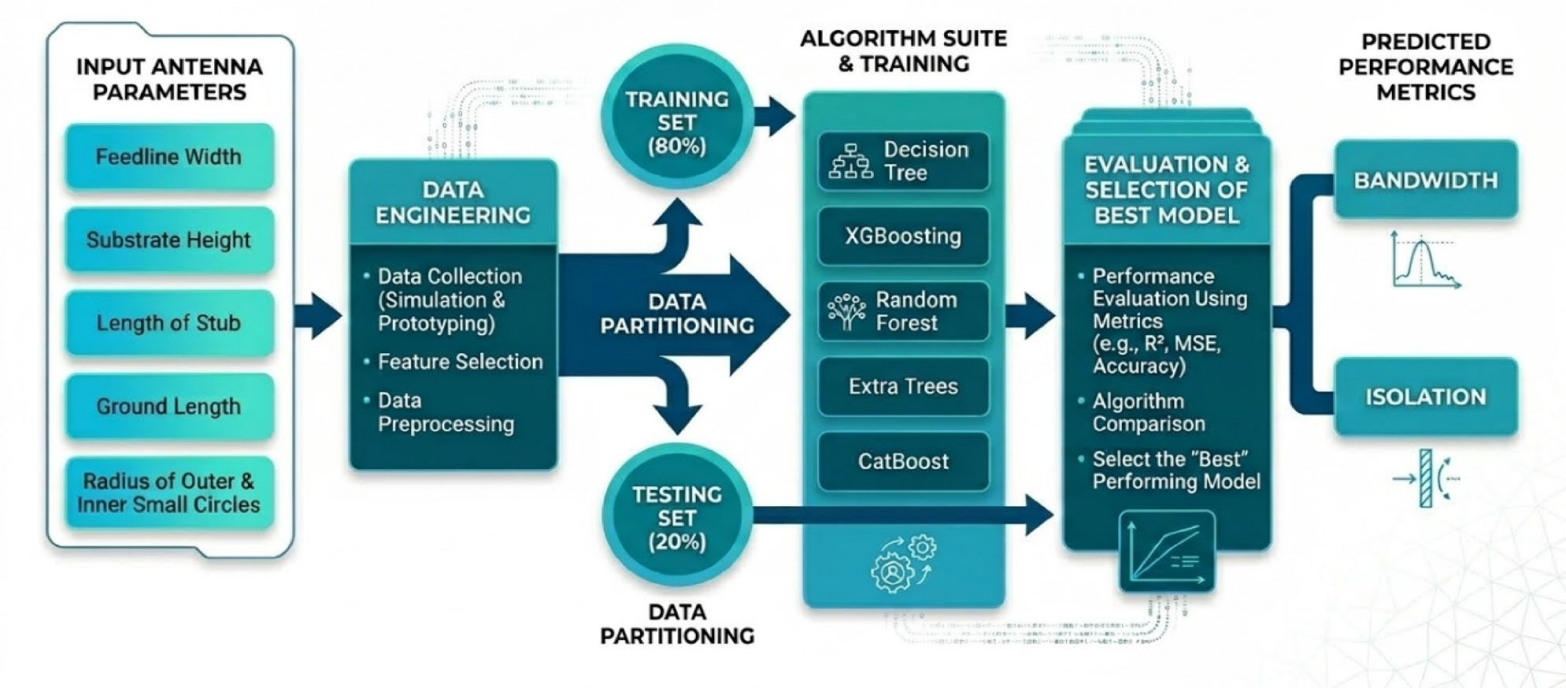
ML workflow for antenna performance prediction.

Building on the dataset analysis presented in Sect. 8.1 and 8.2, this section provides a systematic evaluation of five machine learning algorithms for predicting critical performance metrics of the proposed circular MIMO antenna. Using multiple models ensures the highest possible predictive performance, where regression serves as the fundamental method to model complex relationships between geometric input parameters, such as feedline width, substrate height, stub length, ground length, and circular element radii and key output metrics including bandwidth, isolation, and resonant frequency^48^. By capturing these non-linear design dependencies through advanced regression techniques, the methodology provides engineers with a reliable data-driven framework for optimizing circular MIMO antenna configurations. As illustrated in Fig. 19, the proposed ML pipeline begins with simulation-based data collection, feature selection, and preprocessing, followed by dataset partitioning into training (80%) and testing (20%) subsets. Five algorithms, including Decision Tree, XGBoosting, Random Forest, Extra Trees, and CatBoost, are trained in parallel and evaluated based on R², MSE, and prediction accuracy, with the best-performing model selected to predict bandwidth (BW) and isolation as the two critical performance metrics for THz MIMO communication applications.

Random Forest is an ensemble learning method that combines multiple decision trees to generate reliable predictions for both regression and classification tasks. Each tree is trained on a bootstrap-sampled subset of the data, with random feature selection applied at each split, and final outputs obtained through averaging (regression) or majority voting (classification)^49^. This approach reduces overfitting, improves generalization, and enhances prediction accuracy. Extra Trees (Extremely Randomized Trees) further increases randomness in the tree-building process. Unlike Random Forest, it uses the entire dataset and selects feature splits randomly without optimization^50^. This higher degree of randomness can improve performance in high-dimensional problems such as antenna design optimization, while also reducing overfitting and computational cost by eliminating split selection procedures. Decision Trees are non-parametric supervised learning models that construct hierarchical, tree-like structures consisting of nodes and branches. By recursively splitting data based on feature values, they produce interpretable flowchart-like decision rules that lead to final predictions at the leaf nodes^51^. Their strong interpretability makes them particularly valuable in antenna design, where understanding the relationship between parameters and performance is essential. XGBoost (Extreme Gradient Boosting) builds ensembles of decision trees sequentially, where each tree corrects the errors of the previous one using gradient-based optimization^52^. With built-in regularization and parallel processing, it offers high accuracy, reduced overfitting, and strong computational efficiency, making it well-suited for complex antenna performance prediction tasks. CatBoost (Categorical Boosting) is another gradient boosting method that constructs decision trees iteratively to minimize prediction errors^53^. It builds each new tree to correct the residuals of prior models, resulting in progressively improved predictive performance and high accuracy.

### Hyperparameter optimization procedure for ML models

Hyperparameter optimization is a crucial step in the development of robust machine learning (ML) models, as model performance strongly depends on the appropriate selection of parameters that govern learning behavior, model complexity, and regularization. In this study, all five employed models are systematically tuned using grid search or randomized search in combination with cross-validation to identify optimal configurations that maximize predictive accuracy while minimizing overfitting.

For the Decision Tree regressor, the key hyperparameters include maximum tree depth (max_depth), minimum samples required to split an internal node (min_samples_split), minimum samples at a leaf node (min_samples_leaf), and the splitting criterion (mean squared error or mean absolute error)^51^. These parameters are carefully adjusted to control tree complexity and achieve an optimal trade-off between bias and variance, thereby avoiding overfitting due to excessively deep or overly complex trees. For the XGBoost regressor, the optimized hyperparameters include the number of boosting rounds (n_estimators), learning rate (eta), maximum tree depth (max_depth), subsample ratio (subsample), column sampling ratio (colsample_bytree), and regularization terms (alpha and lambda)^52^. Randomized search with cross-validation is employed to efficiently explore this high-dimensional hyperparameter space while maintaining computational feasibility and ensuring strong generalization performance. For the Random Forest regressor, tuning is performed on parameters such as the number of estimators (n_estimators), maximum depth (max_depth), number of features considered at each split (max_features), minimum samples required for splitting (min_samples_split), and minimum samples at leaf nodes (min_samples_leaf)^49^. These parameters are optimized to enhance ensemble diversity while preserving the strength of individual trees, thereby improving variance reduction and overall predictive stability. For the Extra Trees regressor, a similar set of hyperparameters is optimized, including n_estimators, max_depth, max_features, min_samples_split, and min_samples_leaf^50^. Particular emphasis is placed on controlling the degree of randomness in feature and split selection, which helps reduce variance while maintaining acceptable bias levels, leading to improved generalization capability. For the CatBoost regressor, the tuned hyperparameters include the number of iterations, learning rate, tree depth, L2 regularization coefficient (l2_leaf_reg), and bagging temperature. Additionally, CatBoost’s built-in overfitting detection mechanism is used in conjunction with cross-validation to automatically determine the optimal number of iterations, ensuring both high predictive accuracy and efficient model convergence^53^.

### Performance measurement metrics

The accuracy and efficacy of the five ML models are evaluated using multiple performance metrics that provide comprehensive insight into model behavior. R-squared (R²) and explained variance score quantify the model’s ability to capture dataset variability, while mean squared error (MSE), root mean squared error (RMSE), and mean absolute error (MAE) measure prediction error magnitudes^54^. Error-based metrics (MAE, RMSE) reflect prediction accuracy, whereas variance-based metrics (R², variance score) assess explanatory power. Together, these metrics provide a robust evaluation framework for circular MIMO antenna optimization.

Mean Absolute Error (MAE) computes the average absolute difference between predicted and actual values, treating all errors equally and providing a straightforward accuracy measure^55^, as given in Eq. (10). Mean Squared Error (MSE) calculates the average squared difference between predicted and actual values, expressed in squared units of the target variable^56^. It penalizes larger errors more strongly, making it sensitive to outliers, as shown in Eq. (11). Root Mean Squared Error (RMSE) is the square root of MSE, providing error values in the same units as the target variable for easier interpretation^57^. It also penalizes larger deviations more heavily, as expressed in Eq. (12). R-squared (R²) measures the proportion of variance in the dependent variable explained by the model, with values close to 1 indicating strong performance, while values near 0 indicate poor fit^58^. It can also become negative when the model performs worse than a mean-based predictor, as defined in Eq. (13). The Variance Score evaluates how well the model captures data variability, with values close to 1 indicating strong predictive performance and values near 0 indicating poor fit, as shown in Eq. (14)^59^.

10 $$\:MAE=\:\frac{1}{n}{\sum\:}_{i=1}^{n}\left|\widehat{{y}_{i}}-{y}_{i}\right|$$

Here, $\:\widehat{{y}_{i}}$ and $\:{y}_{i}$ are the actual and projected values, respectively and n is the number of samples.

11 $$\:MSE=\:\frac{1}{n}{\sum\:}_{i=1}^{n}{(\widehat{{y}_{i}}-{y}_{i})}^{2}$$

12 $$\:RMSE=\:\sqrt{MSE}=\:\sqrt{\frac{1}{n}{\sum\:}_{i=1}^{n}{(\widehat{{y}_{i}}-{y}_{i})}^{2}}$$

13 $$R^{2} = {\mathrm{1}}{-}\left( {{\mathrm{SSE}}/{\mathrm{SST}}} \right)$$

In this instance, SST calculates the total variance of the dependent variable by adding the squared differences between the actual and mean values, while SSE indicates the sum of squared differences between the actual and predicted values.

14 $${\mathrm{Variance}}\;{\mathrm{Score}} = {\mathrm{1}} - \frac{{{\mathrm{Var}}\left( {\widehat{{{\mathrm{y}}_{{_{{\mathrm{i}}} }} }} - {\mathrm{y}}_{{\mathrm{i}}} } \right)}}{{{\mathrm{var}}\left( {\mathrm{y}} \right)}}$$

In this case, $\:\widehat{{y}_{i}}$ and $\:{y}_{i}$ respectively, represent the expected and actual values.

## Regression model performance analysis

### Bandwidth prediction in circular MIMO antennas via regression

**Fig. 20:**
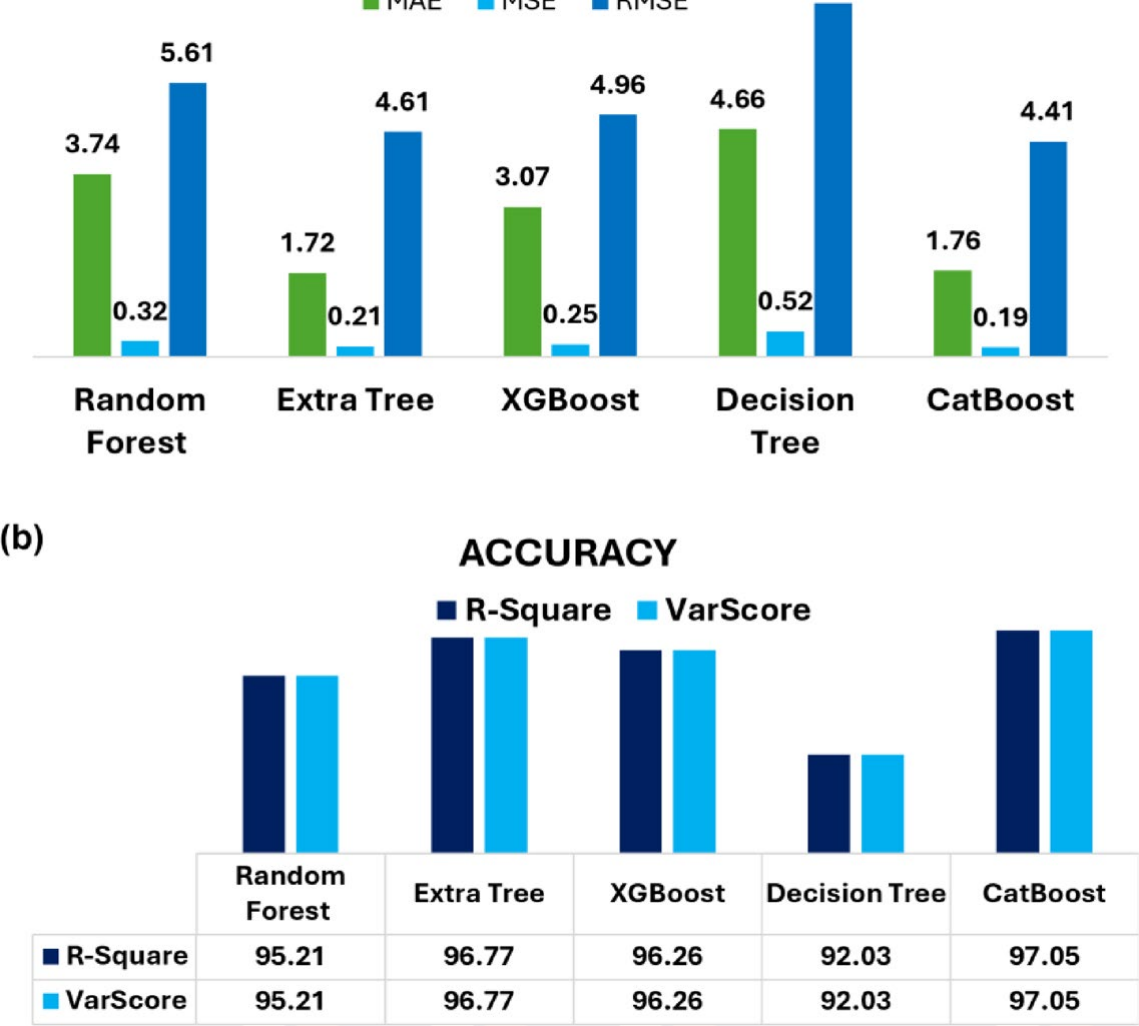
(**a**) Error metric evaluation and (**b**) Regression fit accuracy for bandwidth prediction.

The comprehensive error-based performance evaluation presented in Table 4; Fig. 20 reveals significant performance disparities among five regression models for bandwidth prediction in circular MIMO antennas, assessed through MAE, MSE, and RMSE metrics. CatBoost regressor emerged as the dominant performer, delivering exceptional predictive accuracy with remarkably low error rates of MAE 1.76%, MSE 0.19%, and RMSE 4.41%, demonstrating its superior capability to model complex nonlinear bandwidth relationships with outstanding precision and reliability in THz applications. Extra Tree regressor achieved competitive second-place performance with closely comparable metrics of MAE 1.72%, MSE 0.21%, and RMSE 4.61%, establishing itself as a robust alternative for high-precision bandwidth estimation tasks. Random Forest and XGBoost models delivered moderate prediction accuracy with acceptable error ranges, though their performance remained inferior to the leading ensemble methods. In contrast, the Decision Tree model exhibited the poorest overall performance across all evaluation criteria, with substantially higher error values that clearly demonstrate its unsuitability for complex antenna bandwidth prediction applications demanding high accuracy in the terahertz frequency domain.

**Fig. 21:**
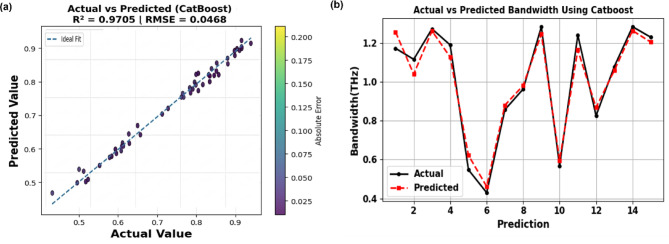
(**a**) CatBoost Regression: Actual vs. Predicted Values (**b**) Bandwidth prediction using CatBoost Regression.

Figure 21a presents the actual versus predicted bandwidth scatter plot for the CatBoost regression model, achieving R² = 0.9705 and RMSE = 0.0468. The predicted values cluster tightly around the ideal fit line with predominantly low absolute errors concentrated in the 0.025–0.075 range, as indicated by the color bar, confirming superior prediction accuracy and minimal deviation across the bandwidth range of 0.5–0.9 THz. The tight alignment of scatter points along the dashed ideal fit line demonstrates CatBoost’s effectiveness in capturing complex nonlinear bandwidth relationships inherent to THz circular MIMO antenna design. Figure 21b further validates CatBoost performance by comparing actual and predicted bandwidth values across 15 test samples spanning 0.4–1.3 THz. The predicted curve closely tracks the actual bandwidth trend, accurately reproducing both the sharp fluctuations and oscillatory variations across all test samples with consistently low error rates. This robust agreement between actual and predicted values confirms that CatBoost successfully generalizes across a wide bandwidth range, demonstrating its suitability for data-driven design and optimization of THz circular MIMO antenna configurations.

**Table 4:** Bandwidth estimation of circular MIMO antennas via regression models.

Algorithms	MAE (%)	MSE (%)	RMSE (%)	*R*^2^ (%)	Var Score (%)
Random Forest	3.74	0.32	5.61	95.21	95.21
Extra Tree	1.72	0.21	4.61	96.77	96.77
XGBoost	3.07	0.25	4.96	96.26	96.26
Decision Tree	4.66	0.52	7.24	92.03	92.03
CatBoost	1.76	0.19	4.41	97.05	97.05

### Isolation estimation using regression models

**Fig. 22:**
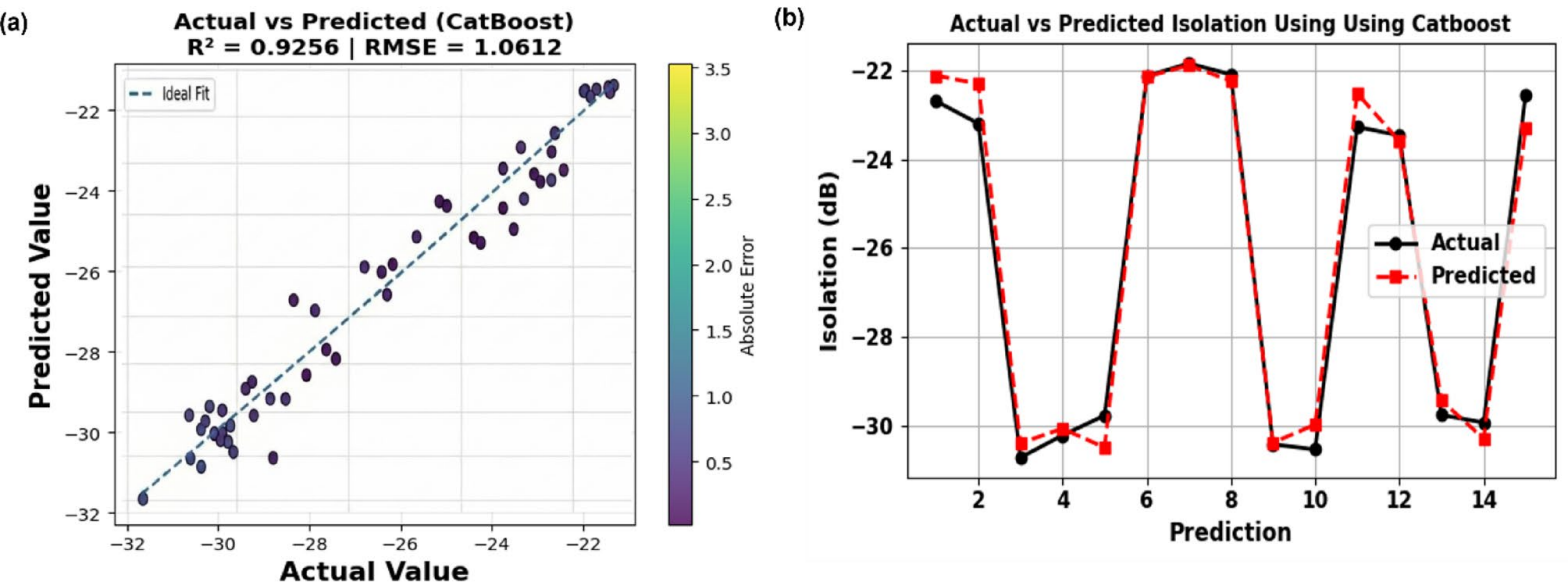
(**a**) Actual vs. Predicted results (CatBoost) (**b**) Isolation prediction performance.

In circular MIMO antenna systems, data-driven isolation prediction leverages advanced machine learning techniques to model mutual coupling effects between antenna elements, enabling faster optimization compared to conventional full-wave simulations. As presented in Table 5, the CatBoost regression model demonstrates strong predictive performance for isolation estimation, achieving MAE = 0.8003, MSE = 1.1262, and RMSE = 1.0612. In addition, the model attains a high coefficient of determination (R² = 0.9256) and variance score (92.57%), indicating excellent accuracy and reliability in capturing the underlying relationship between design parameters and isolation performance. This indicates nearly perfect model reliability. Throughout the crucial 3–8 THz frequency band, Fig. 22 shows a remarkable agreement between the predicted and actual isolation values for the proposed circular MIMO topology, with consistent predictive accuracy maintained throughout the − 22 dB to − 30 dB isolation range, further validating the efficacy of the model. This robust correspondence across diverse validation scenarios underscores the CatBoost algorithm’s superior capability to capture complex electromagnetic interactions inherent in terahertz circular MIMO arrays, positioning it as an invaluable predictive framework for optimizing next-generation 6G communication system designs with enhanced computational efficiency and reliable performance forecasting.

**Table 5:** Isolation estimation for proposed circular MIMO antennas using CatBoost model.

Algorithms	MAE	MSE	RMSE	*R* ^2^	Var Score (%)
CatBoost	0.8003	1.1262	1.0612	0.9256	0.9257

### Performance assessment of CatBoost via K-fold cross-validation

**Fig. 23:**
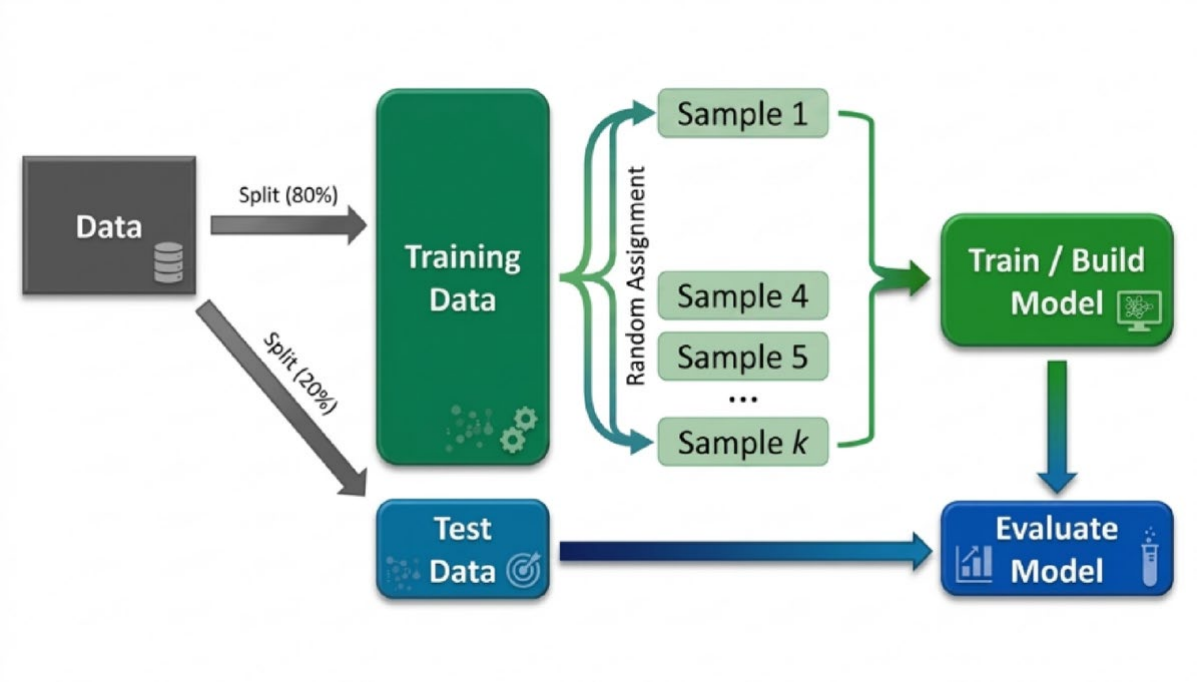
Methodology and workflow of K-Fold cross-validation technique.

K-fold cross-validation is a widely adopted and statistically robust technique for evaluating the generalization performance of machine learning models, particularly when working with limited datasets, such as in antenna design problems where data generation relies on computationally expensive full-wave electromagnetic simulations. In this method, the complete dataset is first divided into K approximately equal and mutually exclusive subsets, known as folds, ensuring that each sample belongs to exactly one-fold^66^. The model is then trained and validated over K iterations, where in each iteration K–1 folds are used for training and the remaining fold is used for validation. This process ensures that every fold is used as the validation set exactly once, allowing all samples to contribute to both training and evaluation in a balanced manner. The final performance is obtained by averaging the evaluation metrics across all iterations, providing a more reliable and variance-reduced estimate compared to a single train–test split. As illustrated in Fig. 23, the dataset is first divided into 80% training data and 20% independent test data, with the test set reserved exclusively for final evaluation. Within the training portion, the data is further partitioned into K folds for cross-validation.

 In this study, 5-fold cross-validation is adopted, where the 240 available samples are partitioned into five equal subsets. In each iteration, one-fold is used as the validation set while the remaining four folds are used to train the machine learning (ML) model. Prior to training, all input features are normalized to enhance numerical stability and ensure efficient convergence of the optimization process. The model parameters are then initialized and trained using the selected learning algorithm for a predefined number of epochs or iterations. Model performance is evaluated on each validation fold using standard regression metrics, including RMSE, MAE, and R². After completing all five iterations, the final performance is obtained by averaging the evaluation results across all folds, thereby reducing the impact of random data splitting and providing a statistically reliable estimate of model generalization. In this work, the proposed Cat Boost regression model is evaluated under the same 5-fold cross-validation framework to ensure robustness and consistency of predictions. The dataset is equally divided into five parts, with each fold serving once as the validation set while the remaining folds are used for training. The aggregated results across all folds demonstrate stable and reliable model behavior. Specifically, the R² values range from 0.8858 to 0.9273 across different folds, indicating strong predictive capability as shown in Table 6. Among these, Fold 4 yields the best performance with the lowest MAE of 0.0487 and the highest R² of 0.9273, whereas Fold 3 shows comparatively lower performance with an R² of 0.8858. Despite these variations, the overall low deviation across folds confirms that the model maintains consistent predictive accuracy and exhibits strong generalization ability with minimal overfitting.

**Table 6:** Cross-validated performance metrics and generalization analysis.

Fold (K)	MAE	MSE	RMSE	*R* ^2^
1	0.0521	0.0047	0.0686	0.9122
2	0.0638	0.0061	0.0781	0.8954
3	0.0694	0.0068	0.0825	0.8858
4	0.0487	0.0043	0.0656	0.9273
5	0.0559	0.0053	0.0728	0.9064
Mean	0.0580	0.0054	0.0735	0.9054

## Limitations of this research

The present study is based on CST electromagnetic simulations combined with machine learning-based predictions, without experimental validation. While the results indicate strong performance, practical implementation of the proposed antenna may face fabrication-related challenges such as alignment accuracy, dimensional tolerances, and material selection, all of which can significantly affect performance at THz frequencies. Even minor geometric deviations or manufacturing imperfections may lead to frequency shifts and bandwidth degradation. In addition, the model is primarily trained and evaluated within the sampled design space, and its extrapolation capability beyond the training distribution has not been extensively investigated. Although the simulation dataset has been expanded from 190 to 240 samples to improve coverage and model robustness, there remains a potential risk of overfitting, where the model may learn dataset-specific patterns rather than fully generalizable relationships, which could affect performance on unseen configurations. Therefore, while the simulation and ML results are promising, establishing practical validity ultimately requires experimental verification. Prototype fabrication and measurement-based characterization are essential next steps to bridge the gap between simulated and real-world performance, and will be systematically addressed in future work to comprehensively validate the proposed circular MIMO antenna design.

## Comparative analysis with state-of-the-art

The performance of the Proposed circular MIMO antenna is thoroughly compared in this section to the most recent published state-of-the-art terahertz MIMO antenna designs, as shown in Table 7. This comparison highlights the proposed antenna’s competitive advantages across pivotal performance metrics that are necessary for upcoming THz and 6G communication systems.

**Table 7:** Comparative evaluation of proposed circular MIMO antenna with state-of-the-art works.

Ref.	Resonance (THz)	Ports	Antenna Size (µm²)	Gain (dB)	Efficiency (%)	Isolation (dB)	Dielectric Material	BW (THz)	ECC, DG (dB)	ML
^60^	0.445, 0.540	4	2490 × 1600	7.9 6.6	81.9, 83.36	<−20	Polyimide	0.05, 0.04	0.0001,10	Yes
^61^	0.4935	2	600 × 300	3.9	79.16	−52.86	Polyimide	0.435	0.1,10	No
^62^	0.395 0.629	4	1200 × 1000	5.01, 5.17	82,92.48	<−20	Polyimide	0.0095 0.024	0.0125, 10	No
^63^	2.2	2	105 × 100	4.4	92	−20	Polyimide	0.78	0.006, 9.9998	No
^64^	0.514	2	600 × 300	5.49	85.24	<−25	Polyimide	0.4	0.015, 9.99	No
^65^	6	2	40 × 20	3.99	90.17	−30.41	Si$\:{o}_{2}$	0.83	0.00000041,10	No
^62^	1.89	2	38 × 25	7.23	74.5	−22.26	Si$\:{o}_{2}$	1.59	0.00000000156, ≈ 10	No
^15^	4.99, 8.831	4	34 × 34	11.89	88.90	−27.34	Polyimide	5.941	0.001, 9.994	Yes
^23^	6.6, 8.2	2	108 × 84	14.59	96	−31	Polyimide	2.5	0.00016,9.9998	Yes
This work	5.55	2	130 × 70	8.038	85.64	−23	Polyimide	1.30	0.0035,9.985	Yes

Regarding bandwidth performance, the proposed design achieves a wide bandwidth of 1.30 THz at 5.55 THz, outperforming several narrowband counterparts such as^60^ (0.05–0.04 THz)^62^, (0.0095–0.024 THz), and^61^ (0.435 THz). Although^62^ reports a slightly higher bandwidth of 1.59 THz, it suffers from lower radiation efficiency (74.5%). In contrast, the proposed antenna maintains a high efficiency of 85.64%, providing a better balance between wideband operation and radiation performance, which is essential for THz communication systems. In terms of gain, the proposed antenna achieves 8.038 dB, which is significantly higher than^61^ (3.9 dB) and^63^ (4.4 dB), with improvements of approximately 4.1 dB and 3.6 dB, respectively. Although slightly lower than high-gain designs such as^23^ (14.59 dB) and^15^ (11.89 dB), the proposed antenna maintains competitive performance while offering advantages in size and bandwidth.

For isolation, the proposed antenna achieves − 23 dB, which is better than or comparable to many reported works such as^63^ (− 20 dB) and^64^ (< − 25 dB), ensuring effective mutual coupling reduction and improved MIMO performance. This level of isolation supports stable signal transmission and enhanced channel independence in THz systems. Radiation efficiency is another key strength, with the proposed design achieving 85.64%, which is higher than^61^ (79.16%) and^60^ (81.9%), while remaining reasonably close to high-efficiency designs such as^23^ (96%) and^65^ (90.17%). This demonstrates that the proposed antenna maintains strong radiation performance without compromising other key parameters.

From a size perspective, the proposed antenna features a compact dimension of 130 × 70 μm² (9100 μm²). Compared to larger designs such as^60^ (2490 × 1600 μm²) and^62^ (1200 × 1000 μm²), the proposed structure achieves significant miniaturization. At the same time, it remains competitive with compact designs such as^63^ (105 × 100 μm²) and^61^ (600 × 300 μm²), while operating at a higher frequency and offering broader bandwidth. Finally, the diversity performance of the proposed circular MIMO antenna is excellent, with an ECC of 0.0035 and a diversity gain of 9.985 dB, both close to ideal values. By combining 8.038 dB gain, 1.30 THz bandwidth, − 23 dB isolation, 85.64% efficiency, and compact size, the proposed design demonstrates a well-balanced and optimized solution for advanced THz applications such as 6G communications, high-speed wireless links, and compact sensing systems.

## Conclusion

This research successfully presents the design, optimization, and machine learning-enhanced prediction of a dual-port compact circular MIMO antenna system engineered for next-generation 6G terahertz applications. The proposed lightweight and miniaturized configuration, comprehensively validated through CST Studio Suite simulations and cross-verified with Ansys HFSS, demonstrates exceptional performance characteristics with resonance at 5.55 THz, return loss of −47 dB, maximum bandwidth of 1.30 THz, inter-port isolation of −23 dB, and peak gain of 8.038 dB per element, establishing performance benchmarks that significantly surpass existing terahertz MIMO designs in the literature. The integration of five regression-based machine learning algorithms revealed CatBoost as the most accurate predictive model, achieving R² = 92.56% for isolation prediction, validated through k-fold cross-validation and comprehensive error metrics including MAE and RMSE. To move beyond simulation-based validation, several specific and actionable future directions are identified. First, in terms of fabrication, polyimide-based THz antenna realization presents key challenges such as maintaining ± 1 μm thickness tolerance, ensuring dielectric uniformity, and achieving accurate sub-millimeter feedline patterning. These can be addressed using electron-beam or high-resolution photolithographic techniques, with fabrication success evaluated by targets such as < 2 dB deviation in return loss and < 3 dB variation in isolation compared to simulations. Second, an experimental validation framework is proposed using a THz vector network analyzer, where performance benchmarks include resonance frequency accuracy within ± 0.05 THz, bandwidth agreement within 10%, and gain verification using near-field scanning measurements.

Third, extension to a 4-port circular MIMO configuration is planned, with explicit targets of maintaining isolation below − 20 dB across all port pairs and achieving an envelope correlation coefficient below 0.1 to ensure strong spatial diversity. Finally, dataset scaling beyond 500 samples and integration of physics-informed neural networks are proposed to enhance model generalization and address current interpolation limitations.

## Data Availability

The datasets generated and/or analyzed during the current study are available from the corresponding author upon reasonable request.
